# Integrated single-cell and metabolomic profiling identifies ovarian metabolic differences and endothelial heterogeneity associated with litter-size variation in Hu sheep

**DOI:** 10.1186/s40104-026-01508-6

**Published:** 2026-10-09

**Authors:** Ting Ge, Xiaoyu Huang, Kuiming Su, Yifan Wen, Bo Li, Shaohua Jiang, Fan Zhao, Enping Zhang, Wen Wang

**Affiliations:** 1 Department of Radiology, Tangdu Hospital, Fourth Military Medical University, 569 Xinsi Road, Xi’an, Shaanxi 710038 People’s Republic of China; 2 Key Laboratory of Animal Genetics, Breeding and Reproduction of Shaanxi Province, College of Animal Science and Technology, Northwest A&F University, Yangling, 712100 China

**Keywords:** Endothelial heterogeneity, Hu sheep, Litter size, Ovarian metabolome, Single-cell transcriptomics

## Abstract

**Background:**

Hu sheep are highly prolific, but litter size remains variable among individuals. How ovarian metabolism and somatic-cell heterogeneity are associated with this variation remains unclear. Ovarian metabolites from high-litter-size (HLS) and low-litter-size (LLS) Hu ewes were profiled by liquid chromatography–mass spectrometry and gas chromatography–mass spectrometry. Ovarian cell populations and transcriptional states were characterized by single-cell RNA sequencing. Integrated analyses were used to relate metabolic differences to specific cell populations and predicted cell–cell communication. RT-qPCR and multiplex immunofluorescence were used to examine selected metabolic genes and the endothelial localization of ESM1. Separate cell experiments assessed the effects of endothelial cell-conditioned medium (EC-CM) and recombinant human IGFBP3 on primary ovine granulosa cells (GCs), with TMEM219 knockdown used to evaluate receptor involvement.

**Results:**

HLS ovaries showed higher L-carnitine abundance, whereas several representative sphingomyelin species were more abundant in LLS ovaries (nominal* P* < 0.05). Integrated analysis identified two EC associated genes: *DNAJB4* was downregulated, whereas *NDRG1* was upregulated in HLS ovaries (*P* < 0.05). At the EC level, gene set enrichment analysis showed significant enrichment of glutathione metabolism and oxidative phosphorylation in the HLS group. EC subclustering further associated these transcriptional programs with ESM1⁺ angiogenic endothelial cells (A-ECs). Multiplex immunofluorescence localized ESM1 to VWF-positive vascular structures and showed a significantly larger ESM1-positive area and more VWF/ESM1 double-positive cells in HLS ovaries (*P* < 0.05). Cell–cell communication analysis identified IGFBP3–TMEM219 as a candidate EC–GC interaction. IGFBP3 was detected in EC-conditioned medium, which significantly reduced EdU incorporation and the expression of *CYP11A1*, *FSHR* and *STAR* (*P* < 0.05) in granulosa cells. Recombinant IGFBP3 produced similar responses, and TMEM219 knockdown partially attenuated several of these effects.

**Conclusions:**

This study identified an association between exploratory ovarian metabolic patterns and EC heterogeneity, while functional experiments showed that EC-derived IGFBP3 affected GC function partly through TMEM219. These findings suggest that endothelial state and signaling may be relevant to follicular development, although whether IGFBP3–TMEM219 signaling contributes to litter-size variation in Hu sheep remains to be established.

**Supplementary Information:**

The online version contains supplementary material available at https://doi.org/10.1186/s40104-026-01508-6.

## Background

Hu sheep are an important indigenous breed in China’s mutton sheep industry and are valued for their high prolificacy [1]. Although genetic determinants of prolificacy have been widely studied, reproductive performance still varies among individuals within this breed. In commercial production, a certain proportion of Hu ewes still produce single lambs, which limits ewe productivity and consequently reduces flock profitability [2]. Therefore, increasing litter size and reducing the proportion of single-lambing ewes remain important breeding objectives in Hu sheep, highlighting the need to better understand the biological basis of litter-size variation. Studies on Hu sheep prolificacy have identified major fecundity genes, particularly *BMPR1B* (*Fec*^*B*^), and implicated several ovarian regulatory pathways, including TGF-β, PI3K–Akt, and Wnt signaling [3, 4]. However, although the *Fec*^*B*^ mutation increases ovulation potential and contributes to enhanced prolificacy, litter size remains variable among ewes with the same *Fec*^*B*^ genotype [5], indicating that major-gene effects alone cannot fully explain individual differences in reproductive performance. These observations suggest that downstream physiological processes, including the synthesis and utilization of ovarian metabolites, may represent an additional regulatory layer associated with litter-size variation.

The ovary provides a specialized local environment for follicular development, oocyte maturation and steroidogenesis [6]. These processes depend on coordinated interactions among germ cells, somatic cells and follicular fluid. Within this microenvironment, metabolites act as nutrients and signaling molecules that support oocyte growth, maturation and developmental competence [7, 8]. Cell-to-cell signaling also shapes this microenvironment. For example, oocyte-derived GDF9 and BMP15 regulate glycolysis and cholesterol biosynthesis in granulosa cells, particularly cumulus cells, which in turn supply metabolites required for oocyte development [9]. In addition, endothelial cell-derived FGF18 acts on granulosa cells and promotes apoptosis in sheep [10]. Consistent with this concept, metabolomic profiling of ovarian tissue and follicular fluid has identified metabolic alterations associated with ovarian function and reproductive capacity [11]. In sheep, follicular-fluid metabolomics has shown amino acid, redox and energy metabolism altered with differential reproductive potential [12]. Distinct follicular-fluid metabolic profiles have also been reported in ewes with different litter sizes [13]. However, the metabolic changes underlying litter-size variation and their relationships with specific ovarian cell populations remain poorly understood in Hu sheep.

Metabolomics captures small-molecule changes that reflect ovarian tissue physiology [14, 15]. Liquid chromatography–mass spectrometry (LC–MS) and gas chromatography–mass spectrometry (GC–MS) broaden metabolite coverage and enable characterization of the ovarian metabolic microenvironment. However, tissue-level metabolomic profiles cannot determine which ovarian cell populations produce or respond to the observed changes. Single-cell RNA sequencing (scRNA-seq) provides complementary information by resolving cell types, transcriptional states and inferred pathway activity. Integrating these approaches can therefore associate ovarian metabolic differences with specific ovarian cell populations and their transcriptional states.

Our previous study investigated differences in ovarian somatic cells between Hu ewes with low and high litter sizes and their potential contribution to prolificacy [16]. Here, we employed metabolomics to identify key differential metabolites between low-litter-size (LLS) and high-litter-size (HLS) Hu ewes. We then integrated these data with single-cell transcriptomics to explore their associations with specific ovarian somatic cell populations. Through this integrated analysis, we aimed to characterize ovarian metabolic differences associated with litter-size variation and identify the somatic cell populations and communication networks potentially linked to these differences.

## Materials and methods

### Ethical statement

The present study was approved by the Animal Care and Use Committee of Northwest A&F University, China (Approval No. DK2021113). All methods and experiments were performed in accordance with the relevant guidelines and regulations.

### Sheep management

The experimental Hu ewes were obtained from Yulin Xinzhongsheng Agriculture and Animal Husbandry Development Co., Ltd. (Shaanxi, China). Six estrus Hu ewes (average age: 3.6 years) were divided into two groups based on litter size from three consecutive parturitions. The high litter size group (HLS group, *n* = 3, body weight: 40.16 ± 1.19 kg) consisted of sheep with a litter size ≥ 3, while the low litter size group (LLS group, *n* = 3, body weight: 42.01 ± 0.31 kg) consisted of sheep with a litter size of 1. Sheep weight and production records are shown in Table 1.

**Table 1:** Sheep weight and production records

Sheep	Weight, kg	Litter size	Birth date	Birth weight of lamb, kg
HLS-1	37.87	3	2020-04-05	2.6/2.8/2.6
			2020-11-27	2.9/2.8/2.8
			2021-08-31	2.4/2.8/2.7
HLS-2	40.74	3	2020-04-11	2.3/2.7/2.36
			2020-12-08	2.7/2.9/3.0
			2021-0908	3.0/2.9/2.7
HLS-3	41.88	3	2020-03-30	3.0/2.8/2.9
			2020-11-25	3.1/2.6/2.8
			2021-08-28	2.8/3.0/2.8
LLS-1	41.39	1	2020-04-15	4.3
			2020-11-30	5
			2021-08-28	5.2
LLS-2	42.41	1	2020-04-07	3.9
			2020-12-01	4.1
			2021-09-05	3.8
LLS-3	42.23	1	2020-04-15	4.6
			2020-12-10	4.4
			2021-09-01	4.3

The ram test was used to determine the estrous status. Then six randomly selected ewes were slaughtered within 12 h of estrus. Whole ovary tissue were collected and preserved at −80 °C for subsequent metabolomics, scRNA-seq and tissue validation analysis.

### Metabolome

Sample preparation for both GC–MS and LC–MS analyses followed identical initial procedures. Precisely weighed samples (30 mg) were transferred to 1.5-mL microcentrifuge tubes containing two steel beads. Subsequently, 20 μL of internal standard solution (0.3 mg/mL 2-chloro-L-phenylalanine in methanol) and 600 μL of extraction solvent (methanol/water, 4:1, v/v) were added to each sample. Samples were flash-frozen at −80 °C for 2 min, homogenized at 60 Hz for 2 min, ultrasonicated at ambient temperature (25–28 °C) for 10 min, and incubated at −20 °C for 30 min. Following centrifugation (13,000 × *g*, 4 °C, 15 min), 150 μL aliquots of supernatant were collected using glass syringes, filtered through 0.22-μm membranes, and transferred to autosampler vials.

For GC–MS analysis, 80 μL of methoxyamine hydrochloride (15 mg/mL in pyridine) was added to each vial for derivatization prior to analysis. GC–MS was performed using an Agilent 7890B system (Agilent Technologies, Santa Clara, CA, USA) equipped with a DB-5MS capillary column (30 m × 0.25 mm × 0.25 μm). LC–MS analysis was conducted using an ACQUITY UPLC I-Class PLUS system (Waters Corporation, Milford, MA, USA) coupled to a Q-Exactive quadrupole-Orbitrap mass spectrometer (Thermo Fisher Scientific). Separation was achieved on an ACQUITY UPLC HSS T3 column (100 mm ×2.1 mm, 1.8 μm) maintained at 40 °C.

### Metabolome data preprocessing

The LC–MS raw data were processed through the metabolomics analysis software Progenesis QI v2.3 (Nonlinear Dynamics, Newcastle, UK) for baseline filtering, peak identification, integration, retention time correction, peak alignment, and normalization. The identification of compounds was based on mass-to-charge ratio (*m/z*), secondary fragments and isotope distribution, and qualitative analysis was performed using databases such as the Human Metabolome Database (HMDB), LipidMaps (v2.3), METLIN, and custom libraries. Using Analysis Base File Converter software, the raw GC–MS data were transformed into ABF format. Subsequently, the data were processed with MS-DIAL software for peak detection, deconvolution, alignment, and filtering. The metabolites were annotated utilizing the LUG database (untargeted database of GC–MS data; Lumingbio).

The positive and negative data were combined into one dataset that was imported into the R package ropls. Principal component analysis (PCA) and orthogonal partial least-squares discriminant analysis (OPLS-DA) were carried out to visualize the metabolic alterations among experimental groups, after mean centering (Ctr) and Pareto variance (Par) scaling, respectively. The Hotelling’s T2 region, shown as an ellipse in score plots of the models, defines the 95% confidence interval of the modeled variation. Variable importance on projection (VIP) ranks the overall contribution of each variable to the OPLS-DA model, and those variables with VIP > 1 are considered relevant for group discrimination. In this study, the default 7-round cross-validation was applied, with 1/7 of the samples excluded from the mathematical model in each round avoid overfitting. The differential metabolites were selected based on the combination of a statistically significant threshold of VIP values obtained from the OPLS-DA model and *P* values from a two-tailed Student’s *t*-test on the normalized peak areas, where metabolites with VIP values larger than 1.0 and *P* values lower than 0.05 were considered as differential metabolites. Differential metabolites pathway enrichment used the Kyoto Encyclopedia of Genes and Genomes (KEGG) database.

### Metabolome-scRNA integrative analysis

The detail of ScRNA dimensionality reduction, batch correction, and cell type annotation was described in Method S1 (Additional file 1). Differential expression between the HLS and LLS groups was analysed using the FindMarkers function in Seurat. *P*-values were adjusted using the Benjamini–Hochberg method, and genes with an adjusted *P* < 0.05 were considered differentially expressed. Differential metabolites were identified using the criteria of nominal* P* < 0.05 and VIP > 1. The Pearson correlation algorithm was applied to calculate the correlation between the expression levels of differential genes and the intensity of response of differential metabolites.

### Identification of endothelial cell subtypes

For endothelial cells, subset analyses were conducted to achieve higher resolution. After subset extraction, RunPCA, FindNeighbors, FindClusters [17], and Run uniform manifold approximation and projection (UMAP) were re-executed to delineate fine-scale substructures. To resolve endothelial cell heterogeneity, six subtypes were identified and annotated based on enriched subtype markers: inflammatory ECs (CXCL9), angiogenic ECs (ESM1), capillary ECs (HOPX), endothelial-to-mesenchymal transition-like ECs (EndMT-like ECs) ECs (COL1A1), arterial ECs (GJA5), and lymphatic ECs (CCL21).

### Bioinformatics analysis of single-cell transcriptome data

#### Differential expression analysis

Differential expression analysis was performed at the cluster level between the HLS and LLS groups. Significantly differentially expressed genes (DEGs) were mapped to their gene IDs.

#### Functional enrichment analysis

Based on these DEGs, KEGG enrichment analyses were conducted using the clusterProfiler R package [18]. To evaluate pathway alterations from a whole-transcriptome perspective, Gene Set Enrichment Analysis (GSEA) [19] was performed. Furthermore, Gene Set Variation Analysis (GSVA) [20] was employed to estimate pathway activity scores for individual cells or samples in a non-parametric manner. Specifically, the single-sample GSEA (ssGSEA) method was adopted with appropriate kernel settings for discrete data to generate a pathway activity matrix. Differential analysis of pathway activities between groups or cell types was subsequently performed using the limma package [21], with an adjusted *P*-value < 0.05 considered statistically significant.

#### Pseudotime trajectory analysis

Trajectory analysis was performed using Monocle 2 [22]. The resulting branch-dependent genes were visualized using the ClusterGVis R package [23] (Additional file 1, Method S2). Genes showing dynamic expression patterns along the pseudotime trajectory were grouped into clusters and subjected to Gene Ontology (GO) enrichment analysis.

#### Intercellular communication analysis

For downstream analyses requiring human-derived reference resources, ovine genes were first mapped to their human homologs using Ensembl BioMart, and only successfully mapped genes were retained, following a recently reported strategy for Hu sheep single-cell data [24]. CellChat [25], using the human ligand–receptor database (CellChatDB.human), was applied for global network inference and pathway-level comparisons. NicheNet [26] for causal inference from sender ligands to receiver target genes (Additional file 1, Method S3).

#### Metabolic analysis

Following ortholog mapping, metabolic pathway activity scores were calculated using scMetabolism [27] based on KEGG-derived metabolic gene sets. Relative metabolic fluxes were estimated using single-cell flux estimation analysis (scFEA) [28]. Overall differences in metabolic activity across cell types or between HLS and LLS groups were visualized. To infer metabolic reaction rates at single-cell resolution, scFEA was employed to estimate the fluxome. scFEA reconstructs the metabolic network as a factor graph comprising metabolic modules and intermediate metabolites. By incorporating flux balance constraints at the whole-cell level, it utilizes a graph neural network optimization framework to map enzyme-encoding gene expression to metabolic module fluxes, yielding relative flux distributions and metabolite imbalance scores for each cell. The flux results were imported into Seurat for downstream statistical analysis and visualization. Metabolic module fluxes were standardized across cells (z-score), and aggregated comparisons were performed by cell type and group.

### Functional assessment of endothelial derived IGFBP3 in granulosa cells

#### Multiplex immunofluorescence staining

Paraffin-embedded ovarian sections from HLS and LLS Hu ewes were subjected to multiplex immunofluorescence staining for ESM1 and endothelial markers. Sections were incubated with primary antibodies against VWF (HUABIO, HA722833; 1:100), and ESM1 (Affinity Biosciences, DF14935; 1:100), followed by signal amplification using tyramide signal amplification reagents. Detailed staining procedures are provided in Method S4 (Additional file 1). Whole-slide images were acquired using an Olympus VS200 scanner. ESM1-positive areas and VWF/ESM1 double-positive cells were quantified using HALO and Image-Pro Plus software, respectively. Two non-overlapping fields per animal were analyzed, and each animal was considered as an independent biological replicate.

#### RT-qPCR of metabolic genes in ovarian tissue

Ovarian tissues were obtained from HLS and LLS Hu ewes used for the experiment (*n* = 3 animals per group). *NDRG1*, *GCLC* and *ATP5F1A* was determined by reverse-transcription quantitative PCR (RT-qPCR; Method S5). Primer sequences are listed in Table 2.

**Table 2:** Primer sequences

Name	Primer	Sequence (5′→3′)
*GAPDH*	Forward	ACAGTCAAGGCAGAGAACGG
	Reverse	CCAGCATCACCCCACTTGAT
*NDRG1*	Forward	ACATCCTCACTCGCTTTGCT
	Reverse	TTGGCAAATACTGGGGCCTTG
*CYP11A1*	Forward	TCCGTGACCCTGCAGAGATA
	Reverse	TAGATGGCCACTTGCACCAG
*FSHR*	Forward	CTGGCCATCACTGGGAACAT
	Reverse	ACTGGCTTTTGGTGTGGACA
*STAR*	Forward	CTGGCTGGAAGTCCCTCAAG
	Reverse	AATCCACTTGGGTCTGCGAG
*GCLC*	Forward	GGAGGAAACCAAGCGCCAT
	Reverse	CTTGACGGCGTGGTAGATGT
*ATP5F1A*	Forward	GTCTGTGTCCCGTGTTGGAT
	Reverse	ACTCAGTCAAACGCACACCA
*TMEM219*	Forward	ACGGTCCAGACAGGAACAAGA
	Reverse	CTGAGCAGGATATGGGTGGC

#### Preparation of endothelial cell-conditioned medium

Ovarian microvascular endothelial cells (HOMECs; Cellverse, hum-icell-f050) were used to generate endothelial-cell conditioned medium (EC-CM), due to the limited availability and technical challenges associated with isolating sheep ovarian endothelial cells. After serum-free conditioning, EC-CM was collected for IGFBP3 quantification by ELISA (ZCIBIO, ZC-35444). IGFBP3 secretion was normalized to total cellular protein content. Detailed procedures and ELISA result are provided in Method S6 (Additional file 1).

#### Experimental validation and endothelial-granulosa cell communication

Sheep primary ovine granulosa cells (Additional file1, Method S7) were used for functional validation experiments. Granulosa cells were subjected to two treatment schemes. In the first treatment scheme, granulosa cells were cultured for 48 h in either serum-free medium (CON group; *n* = 3) or HOMEC-derived conditioned medium (EC-CM group; *n* = 3).

In the second scheme, cells were transduced with pLKO.1 carrying either a non-targeting short hairpin RNA (shNC) or an shRNA targeting ovine TMEM219. Three candidate shRNAs were initially screened by RT-qPCR, and the construct producing the greatest reduction in TMEM219 expression was selected for subsequent experiments (Fig. S3E). To determine whether IGFBP3 could reproduce the effects of EC-CM and whether TMEM219 contributed to these responses, primary ovine granulosa cells transduced with shNC or shTMEM219 were treated with recombinant human IGFBP3 (rhIGFBP3). Human IGFBP3 was used to match the species origin of the HOMEC-derived conditioned medium. Following transduction, cells were assigned to four groups according to TMEM219 knockdown and rhIGFBP3 (Sino Biological, 10430-H07H) treatment: shNC, shNC + rhIGFBP3, shTMEM219 and shTMEM219 + rhIGFBP3. Each group included three independent biological replicates (*n* = 3). rhIGFBP3 was used at 0.5 μg/mL, and all treatments lasted 48 h.

Granulosa-cell proliferative activity was assessed using an EdU incorporation assay (Additional file 1, Method S8). The mRNA expression of *CYP11A1*, *FSHR* and *STAR* was measured by RT-qPCR (Additional file 1, Method S5); and the protein abundance of IGFBP3 and TMEM219 was determined by Western blotting (Additional file 1, Method S9). Each group included three independent biological replicates.

### Statistical analysis

Metabolite levels between the LLS and HLS groups were compared using an unpaired two-tailed Student’s *t*-test, nominal *P* < 0.05 indicate significant difference. For single-cell differential expression analysis, *P*-values were adjusted using the Benjamini–Hochberg method, with adjusted *P* value < 0.05 considered significant. Validation data are presented as the mean ± SD from three independent biological replicates and were analysed using Python v3.12.10 (SciPy v1.18.0 and statsmodels v0.14.6). Two-group comparisons used an unpaired two-tailed Student’s *t*-test, whereas four-group comparisons used one-way ANOVA followed by Fisher’s LSD test. qPCR data were analysed using the comparative ΔΔCt method. Unless otherwise stated, *P* < 0.05 was considered statistically significant.

## Results

### Metabolomic profiling and differential metabolite analysis

**Fig. 1:**
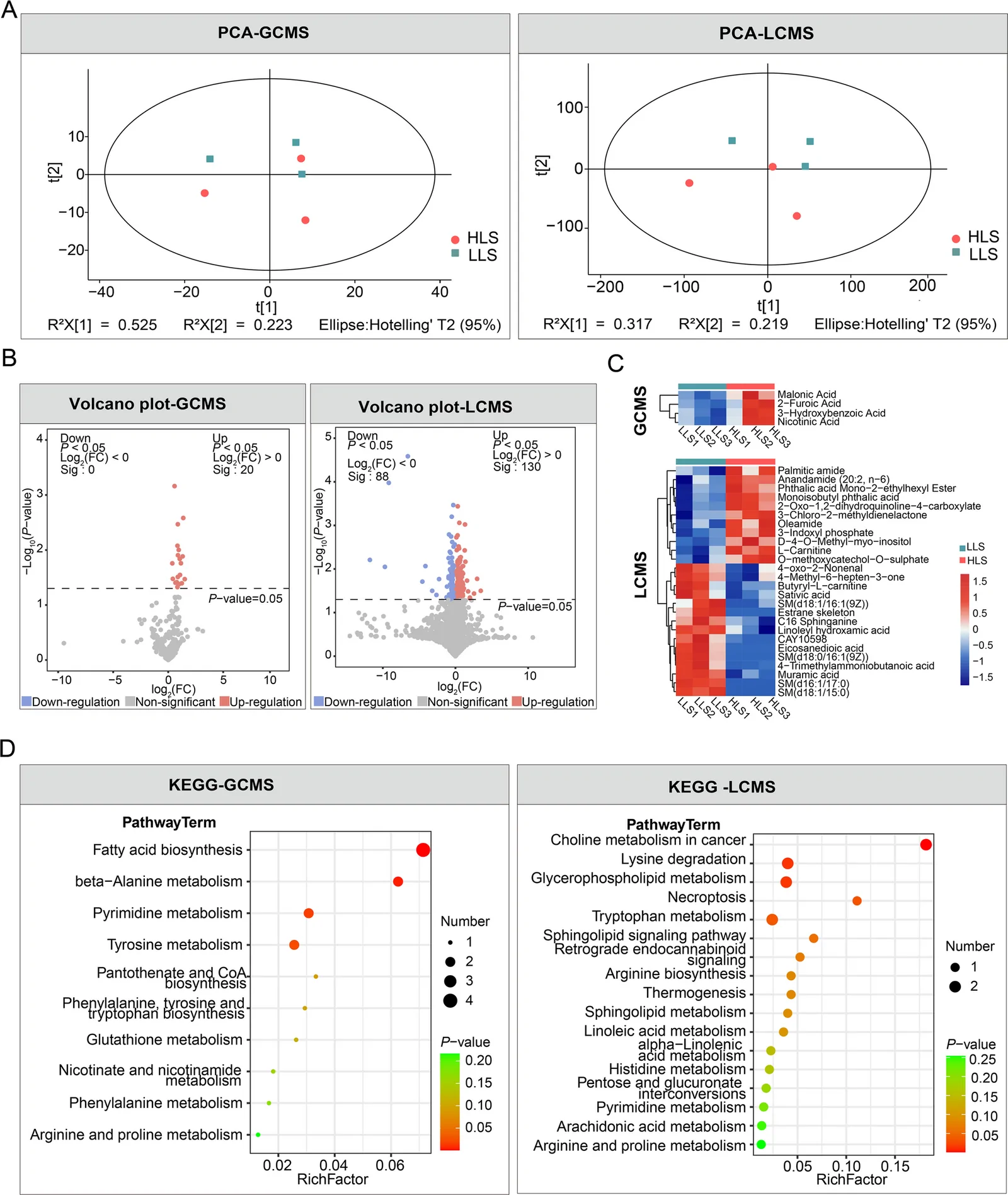
Ovarian metabolome analysis of Hu sheep. **A** PCA analysis of GC–MS and LC–MS. **B** Volcano plots of differential metabolites from GC–MS and LC–MS. **C** Heatmap of representative differential metabolites from GC–MS and LC–MS. **D** KEGG enrichment analysis of differential metabolites from GC–MS and LC–MS

PCA indicated no distinct separation between the HLS and LLS groups in either the GC–MS or LC–MS datasets; however, biological replicates within the same group showed a tendency to cluster (Fig. 1A). Subsequent exploratory screening identified candidate metabolites with different abundance patterns between the two groups. Using VIP > 1 and nominal *P* < 0.05 as the screening criteria, 20 GC–MS metabolites with higher abundance in HLS ovaries were retained as candidates. The LC–MS analysis identified 130 candidates with higher abundance and 88 with lower abundance in HLS ovaries using the same criteria (Fig. 1B; Additional file 2). However, none of the GC–MS or LC–MS candidates remained statistically significant after BH correction. These metabolites were therefore considered hypothesis-generating candidates for subsequent analyses. Then representative differential metabolites between the two groups were visualized via heatmap. Among the representative metabolites detected by LC–MS, L-carnitine was more abundant in HLS ovaries. In contrast, several sphingomyelin (SM) species, including SM(d16:1/17:0), SM(d18:0/16:1(9Z)) and SM(d18:1/16:1(9Z)), were more abundant in LLS ovaries (Fig. 1C; *P* < 0.05). These findings indicate metabolite-specific differences in ovarian energy and lipid metabolism between the two groups.

KEGG pathway analysis provided further insight into the metabolic mechanisms underlying ovarian development in Hu sheep (Fig. 1D). The differential metabolites identified by GC–MS were primarily enriched in “Fatty acid biosynthesis”, “β-alanine metabolism”, “Pyrimidine metabolism” and “Tyrosine metabolism”. These results highlight essential metabolic processes that may regulate ovarian function and reproductive performance. Furthermore, LC–MS data revealed enrichment in 17 metabolic pathways, with the prominent pathways including “Lysine degradation”, “Glycerophospholipid metabolism”, “Necroptosis” and “tryptophan metabolism”.

### Integrated analysis of ovarian metabolites and single-cell transcriptomes in HLS and LLS Hu ewes

**Fig. 2:**
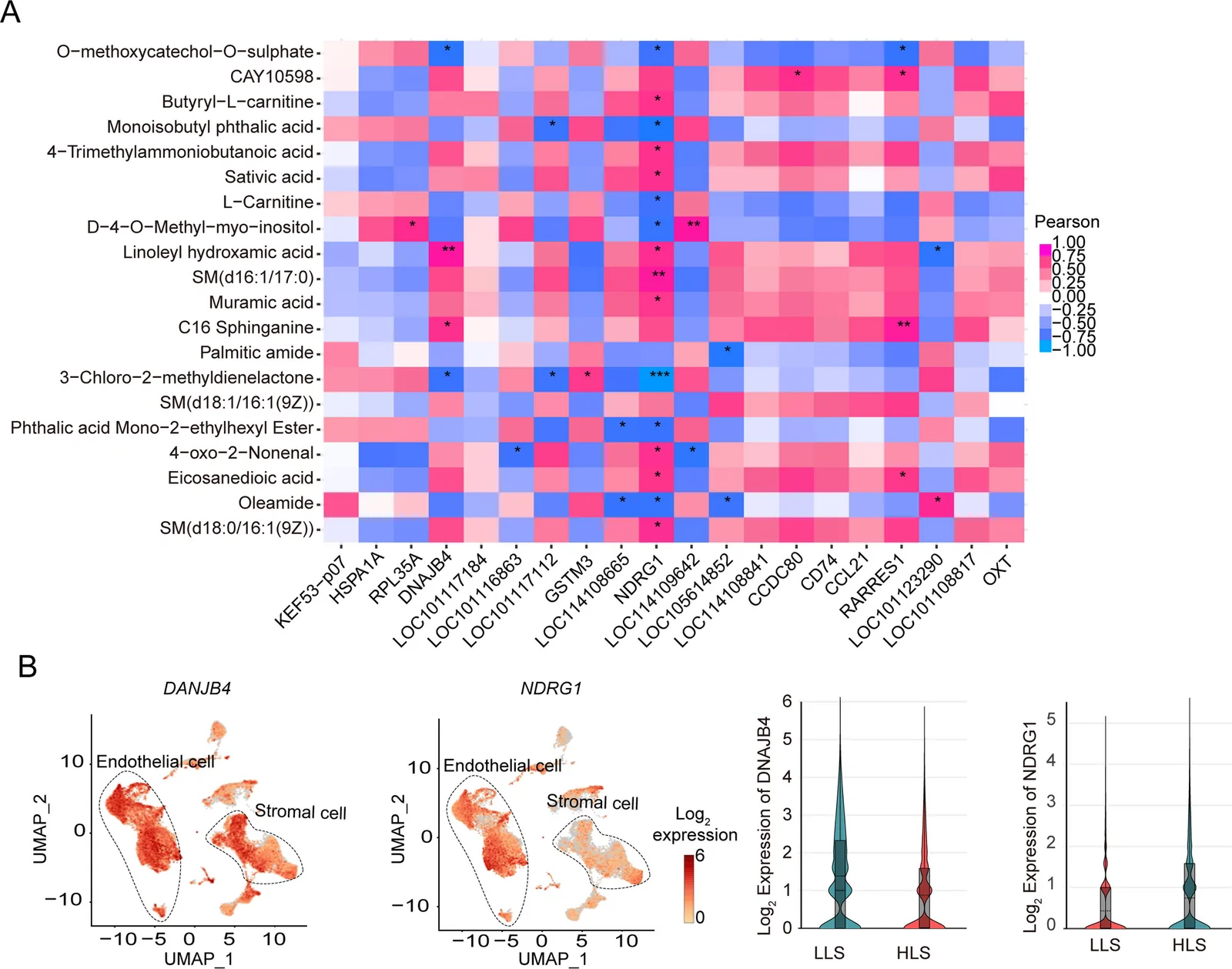
Integrated analysis of metabolome and single-cell transcriptome in HLS and LLS Hu sheep. **A** Correlation analysis of differential metabolites and differential genes. * indicates significant difference. **B** Expression characteristics of *DNAJB4* and *NDRG1*, gene expression characteristics in UMAP and comparison of gene relative expression

To explore potential regulatory connections, we performed an integrated analysis of differential metabolites and genes identified by scRNA-seq. Correlation analysis revealed that *DNAJB4* and *NDRG1* exhibited strong correlations with key differential metabolites (Fig. 2A). Specifically, *NDRG1* displayed significant positive correlations with SM (d16:1/17:0), linoleyl hydroxamic acid, SM (d18:0/16:1 (9Z)), sativic acid, 4-oxo-2-nonenal, 4-trimethylammoniobutanoic acid, butyryl-L-carnitine, eicosanedioic acid, and muramic acid. Conversely, it showed significant negative correlations with 3-chloro-2-methyldienelactone, monoisobutyl phthalic acid, oleamide, D-4-O-methyl-myo-inositol, phthalic acid mono-2-ethylhexyl, L-carnitine, and O-methoxycatechol-O-sulphate. Similarly, *DNAJB4* was positively associated with C16 sphinganine and linoleyl hydroxamic acid, but negatively associated with O-methoxycatechol-O-sulphate and 3-chloro-2-methyldienelactone.

Further examination of cell-type-specific expression using scRNA-seq data indicated that *DNAJB4* was predominantly expressed in endothelial cells (ECs) and stromal cells (SCs), whereas *NDRG1* was specifically enriched in ECs (Fig. 2B). Comparison between groups showed higher *DNAJB4* expression in the LLS group and higher *NDRG1* expression in the HLS group. Consistent with the single-cell data, qPCR analysis of ovarian tissue confirmed that *NDRG1* mRNA expression was significantly higher in HLS ewes than in LLS ewes (Fig. S3A, *P* < 0.05). These results further suggest that metabolic and transcriptional alterations in ECs may contribute to the ovarian differences observed between HLS and LLS ewes, although the underlying mechanisms require further investigation.

### Cell-type-specific functional enrichment and metabolic analysis

**Fig. 3:**
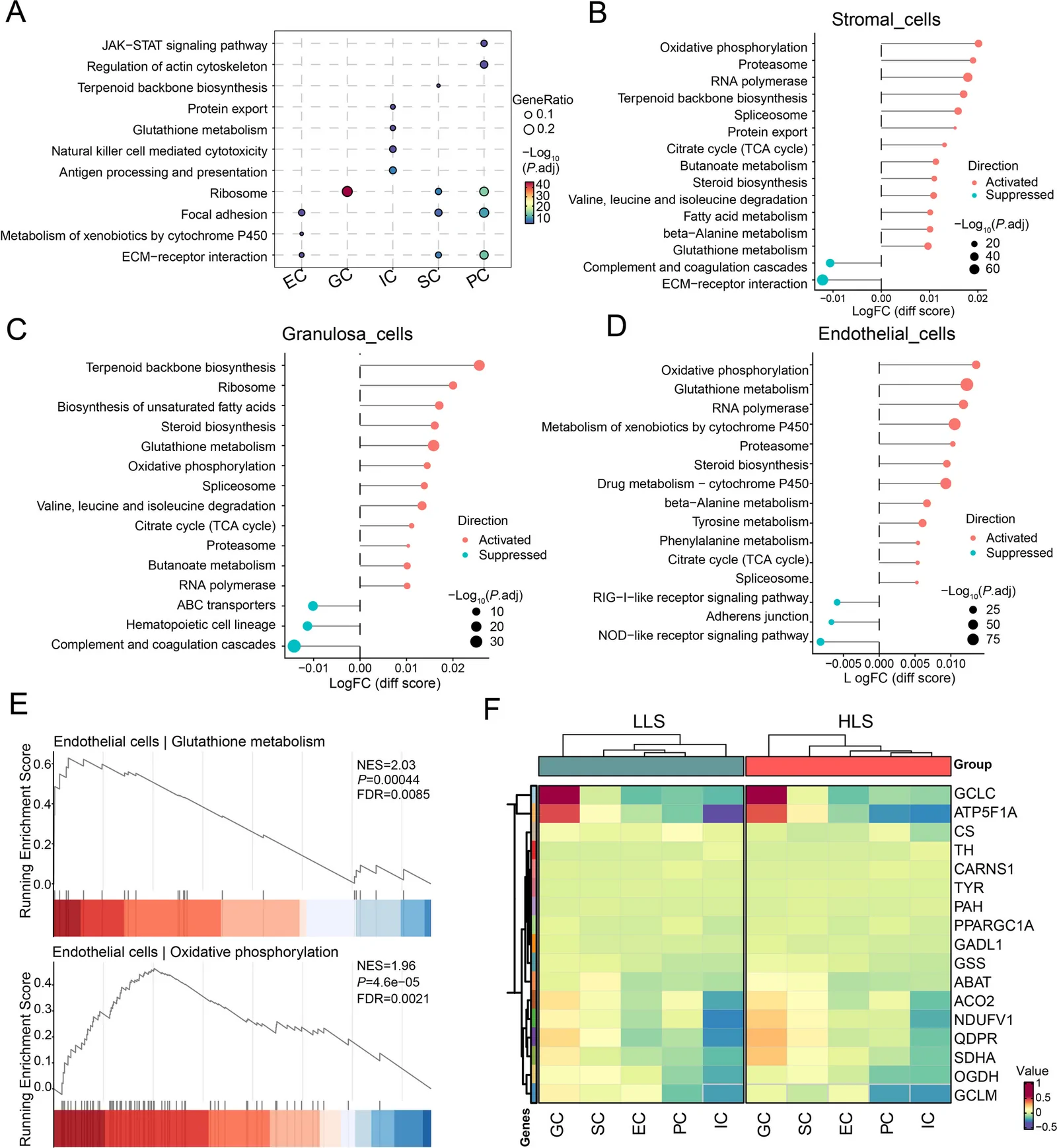
Functional characterization and metabolic pathway analysis of distinct cell subpopulations. **A** Top enriched KEGG pathways across five major cell types. **B**–**D** Differential pathway activity scores in stromal cells (**B**), granulosa cells (**C**), and endothelial cells (**D**) of HLS compared to LLS. **E** GSEA enrichment of Glutathione metabolism (top) and Oxidative phosphorylation (bottom) in endothelial cells. **F** Expression profiles of key metabolic genes involved in oxidative phosphorylation and glutathione metabolism. GC, granulosa cells; SC, stromal cells; IC, immune cells; PC, perivascular cells; EC, endothelial cells, the same abbreviations are used in subsequent figures unless otherwise indicated

Building on our previous identification of GCs as an important somatic cell population associated with Hu sheep prolificacy [16], the present integrative analysis further implicated ECs and SCs in the ovarian differences between the HLS and LLS groups. We therefore examined cell-type-specific functional and metabolic pathway activity across these ovarian somatic cell populations. Following Harmony correction and dimensionality reduction, the major ovarian cell populations were identified for subsequent cell-type-specific analyses (Additional file 1, Method S10). To elucidate the functional heterogeneity underlying ovarian differences between the HLS and LLS groups, we performed pathway enrichment analysis across identified cell types. As shown in Fig. 3A, the “Ribosome” pathway was predominantly enriched in GCs, whereas “Focal adhesion” and “ECM-receptor interaction” were highly enriched in ovarian SCs and PCs, reflecting their structural and supportive roles. We further quantified pathway activity changes within specific cell types. In ovarian SCs (Fig. 3B), pathways related to energy metabolism and protein turnover, such as “Oxidative phosphorylation”, “Proteasome” and “Citrate cycle (TCA cycle)” were significantly activated, whereas structural pathways like “ECM-receptor interaction” were suppressed. A similar trend was observed in GCs (Fig. 3C), where “Terpenoid backbone biosynthesis”, “Ribosome”, and “Steroid biosynthesis” were markedly activated, and these processes are critical for folliculogenesis. Notably, ECs (Fig. 3D) exhibited significant activation of “Glutathione metabolism” and “Oxidative phosphorylation”, suggesting an enhanced capacity for redox regulation and energy production in the HLS group. These findings associate energy- and redox-related transcriptional programmes in ECs with the ovarian differences between HLS and LLS ewes.

To validate these metabolic alterations in ECs, GSEA was conducted. The results confirmed a robust enrichment of “glutathione metabolism” (normalized enrichment score (NES) = 2.03, *P* < 0.01) and “oxidative phosphorylation” (NES = 1.96, *P* < 0.01) (Fig. 3E). Finally, we examined the expression of core genes associated with these metabolic pathways (Fig. 3F). The heatmap visualization revealed differential expression patterns for genes involved in glutathione synthesis (e.g., *GCLC* and *GSS*) and mitochondrial function (e.g., *ATP5F1A*, *ACO2* and *SDHA*) between the two groups. Overall, these genes showed higher expression in ECs from the HLS group than in those from the LLS group. Consistent with this pattern, qPCR analysis of ovarian tissues showed that *ATP5F1A* expression was significantly higher in HLS than that in LLS (*P* = 0.018), while *GCLC* showed a higher expression trend in HLS (Fig. S3A, *P* = 0.061).

### Subpopulation heterogeneity and metabolic trajectory analysis of ovarian ECs

**Fig. 4:**
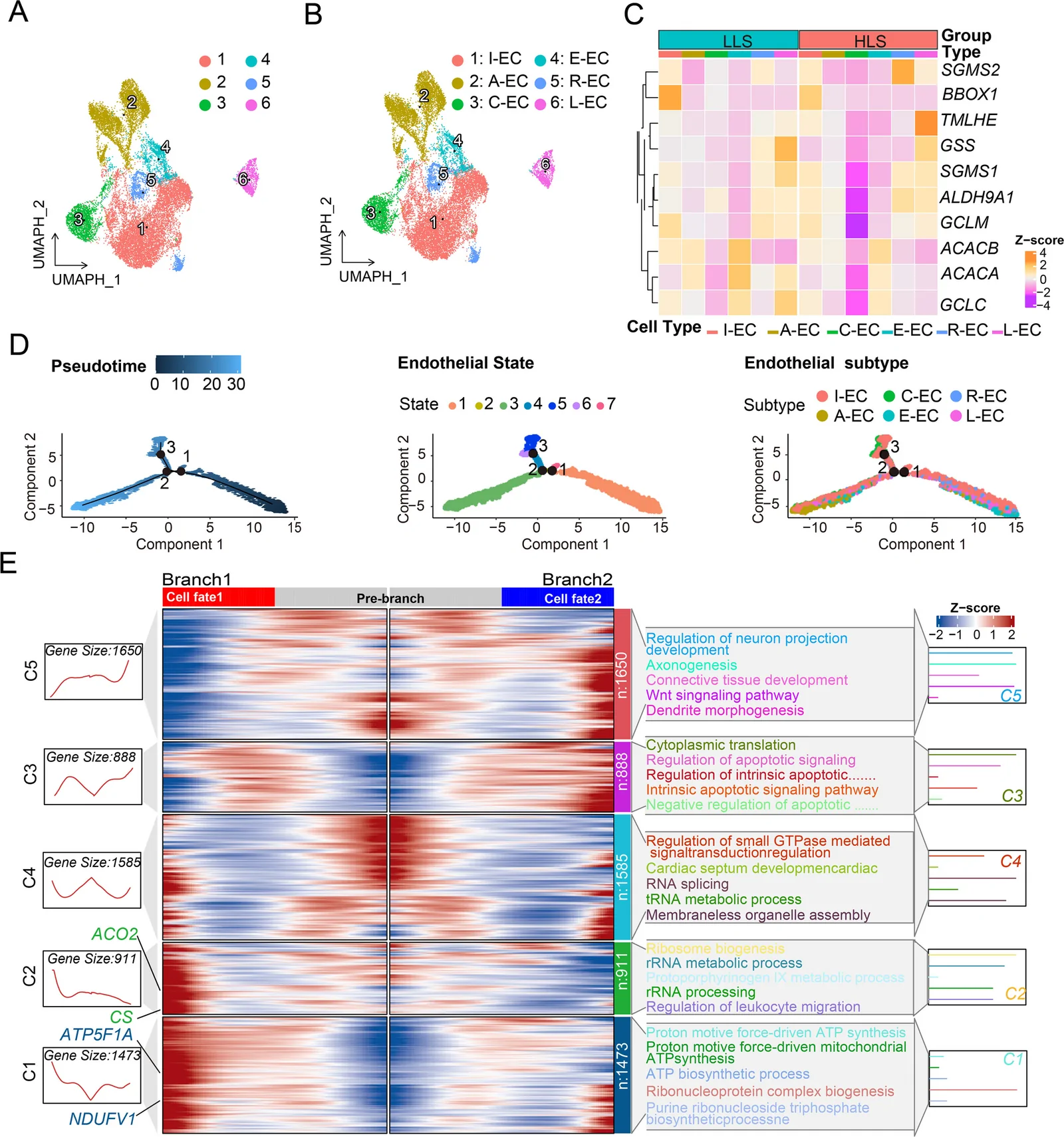
Endothelial cell heterogeneity and pseudotime dynamics in Hu sheep ovaries. **A** and **B** UMAP plots of endothelial cell subclusters colored by cluster ID (**A**) and annotated cell type (**B**). **C** Differential expression of key metabolic genes across different endothelial subtypes and groups. **D** Pseudotime trajectory of endothelial cells, coloured by pseudotime, endothelial state and endothelial subtype. **E** Branched heatmap showing genes with dynamic expression patterns along two endothelial differentiation branches. Gene modules are grouped into five clusters, with representative enriched GO terms shown on the right

To further explore the function of subpopulation heterogeneity and metabolic difference, we performed unsupervised sub-clustering of ECs. As shown in Fig. 4A and B, the ECs were re-clustered into six distinct sub-populations. Endothelial_1 was annotated as inflammatory endothelial cells (Inflammatory_EC, I-EC), characterized by the high expression of the chemokine *CXCL9*. Endothelial_2 specifically expressed the tip-cell marker *ESM1* and was therefore designated as angiogenic endothelial cells (Angiogenic_EC, A-EC). We also identified Endothelial_4 undergoing endothelial-to-mesenchymal transition (EndMT_like_EC, E-EC), marked by the expression of the mesenchymal gene *COL1A1*. The remaining clusters represented specific vascular lineages: Endothelial_3 and Endothelial_5 were identified as capillary endothelial cells (Capillary_EC, C-EC) and arterial endothelial cells (Arterial_EC, R-EC), distinguished by the expression of *HOPX* and *GJA5*, respectively. Finally, Endothelial_6 was defined as lymphatic endothelial cells (Lymphatic_EC, L-EC) based on the canonical marker *CCL21*. To explore the metabolic variations among these subtypes, we analyzed the expression of key genes related to glutathione and lipid metabolism (Fig. 4C). The heatmap revealed significant heterogeneity in metabolic gene expression; notably, genes critical for glutathione synthesis (*GCLC*, *GCLM*, *GSS*) and fatty acid oxidation (*ACACA*, *ACACB*) showed differential expression patterns across subtypes. In particular, the expression levels of these antioxidant and metabolic genes were generally elevated in the HLS group compared to the LLS group within specific EC clusters.

To reconstruct the developmental lineage and functional distinctness of these EC subtypes, we performed pseudotime trajectory analysis (Fig. 4D). The trajectory inferred a branched differentiation path, starting from a root state and bifurcating into two distinct cell fates, including 7 states. Trajectory analysis revealed distinct distribution patterns of endothelial subpopulations across the identified states. I-EC displayed a ubiquitous distribution across all states, suggesting a constitutive role throughout the developmental process. In contrast, A-EC were restricted to a specific temporal window, predominantly appearing in the early phase of state 3. C-EC were mainly concentrated in the later states (states 3, 5, and 6). E-EC occupied a transitional niche, enriched in late state 1 and early state 3. Finally, R-EC were primarily distributed in states 1, 4, and 7 while L-EC were found in states 1, later state of 3, and 4. We next investigated the gene expression dynamics associated with these lineage specifications using branched expression analysis modeling (BEAM) (Fig. 4E). The resulting heatmap identified five gene clusters (C1–C5) with distinct temporal expression patterns. Strikingly, Cluster 1 (C1), which was progressively upregulated along Cell fate 1, was significantly enriched in pathways related to energy metabolism, specifically “ATP biosynthetic process”, “proton motive force-driven mitochondrial ATP synthesis”, and “oxidative phosphorylation”. Key genes in this cluster included *ATP5F1A* and *NDUFV1*. In contrast, Cluster 5 (C5), associated with Cell fate 2, was enriched in “Wnt signaling pathway” and “connective tissue development”.

**Fig. 5:**
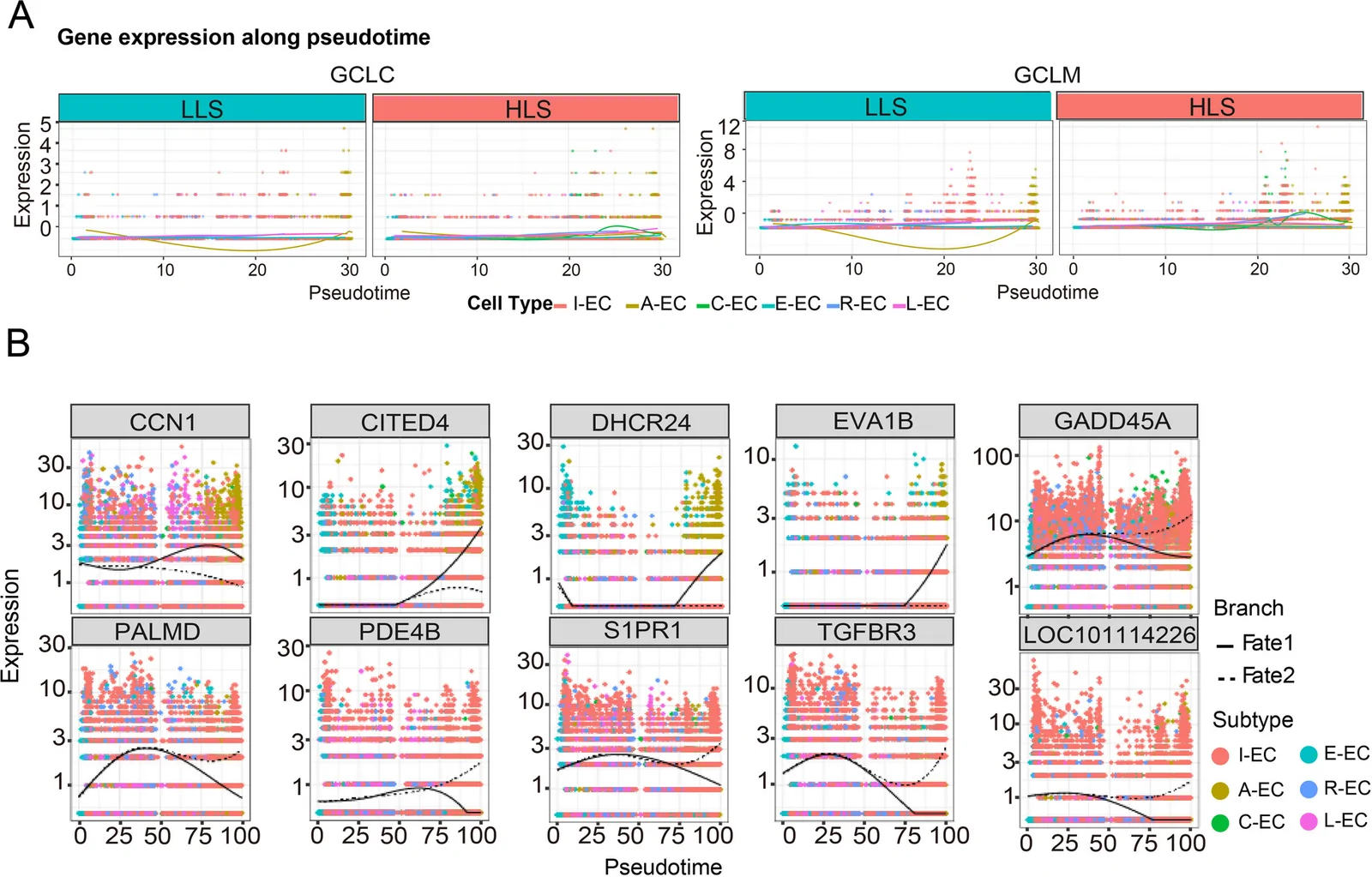
Dynamic expression of glutathione-related and branch-associated genes along endothelial pseudotime. **A** Expression patterns of GCLC and GCLM along endothelial pseudotime in LLS and HLS groups. Cells are coloured by endothelial subtype. **B** Pseudotime expression kinetics of representative genes involved in cell fate determination for Branch 1 (solid line) and Branch 2 (dashed line). Points represent individual cells colored by subtype

Furthermore, we examined the expression dynamics of specific glutathione metabolism genes along the pseudotime trajectory (Fig. 5A). The expression of *GCLC* and *GCLM* exhibited dynamic changes during differentiation. Consistent with our previous findings, the overall expression intensity of these genes was visibly higher and more sustained in the HLS group than in the LLS group across the pseudotime, particularly in the later stages of differentiation. Finally, we identified several branch-dependent transcription factors and marker genes, such as *CCN1*, *CITED4*, and *DHCR24* (Fig. 5B), which may serve as key regulators driving the bifurcation of endothelial cell fates towards different functional states. These findings delineate a high-resolution landscape of ovarian endothelial cell heterogeneity and highlight metabolic reprogramming as a potential contributor to the physiological differences between HLS and LLS groups.

### Single-cell metabolic flux analysis reveals distinct metabolic reprogramming in A-EC subpopulation

**Fig. 6:**
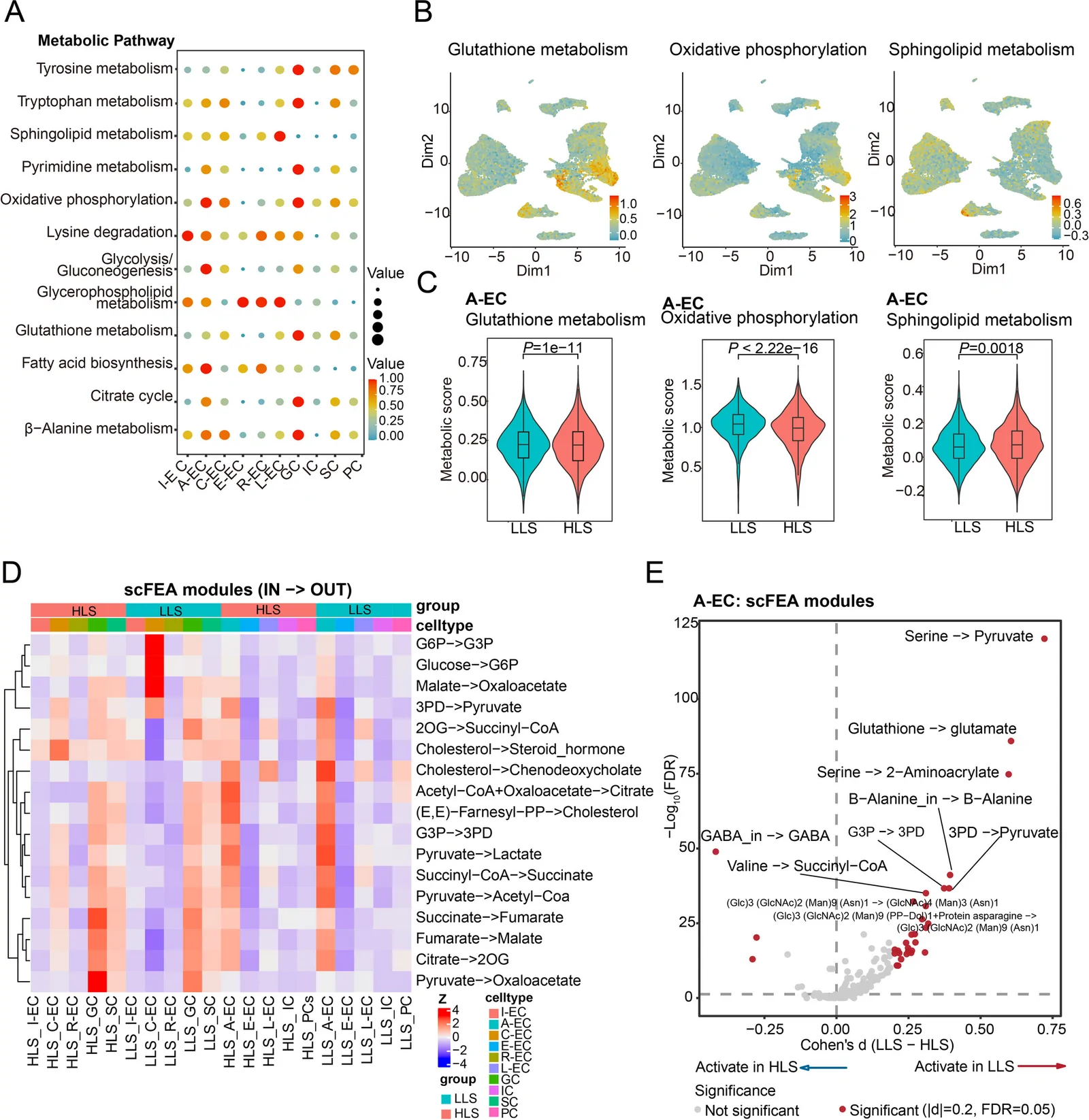
Single-cell metabolic pathway scoring and flux estimation analysis. **A** Average metabolic pathway scores across identified cell subtypes. **B** Feature plots projecting the metabolic scores of glutathione metabolism, oxidative phosphorylation, and sphingolipid metabolism onto the UMAP space. **C** Metabolic scores of glutathione metabolism, oxidative phosphorylation, and sphingolipid metabolism between LLS (blue) and HLS (red) groups specifically in the A-EC subpopulation. **D** Heatmap of scFEA modules across different cell types and groups. **E** Differentially activated metabolic fluxes in A-EC between LLS and HLS groups

To further dissect the metabolic heterogeneity at the single-cell resolution, we evaluated the activitives of the key metabolic pathways identified through metabolomics screening across different cell subpopulations. As shown in Fig. 6A, the metabolic landscape varied significantly among cell types, with endothelial cell subtypes (A-EC) exhibiting distinct enrichment in tyrosine and tryptophan metabolism compared to stromal or immune cells. UMAP visualization further confirmed that the activities of key metabolic pathways, including “Glutathione metabolism”, “Oxidative phosphorylation” and “Sphingolipid metabolism” were spatially heterogeneous and enriched in endothelial cells (Fig. 6B). Hence, we focused on the A-EC subpopulation to investigate group-specific metabolic alterations. Quantitative analysis revealed significant differences in metabolic scores between the two groups. Specifically, A-ECs in the HLS group exhibited altered metabolic signatures in glutathione metabolism (*P* < 0.01), oxidative phosphorylation (*P* < 0.01), and sphingolipid metabolism (*P* < 0.01) compared with those in the LLS group (Fig. 6C).

The scFEA-predicted flux map showed clear cell-type-specific patterns, providing deeper insights into intracellular metabolic dynamics (Fig. 6D). The heatmap of predicted metabolic fluxes displayed clear cell-type-specific patterns, with the A-EC cluster exhibiting unique flux signatures in glucose and TCA cycle-related reactions such as “Pyruvate→ Acetyl-CoA”, and “Citrate→ 2OG”. Finally, differential flux analysis (Fig. 6E) highlighted specific metabolic reactions that were significantly altered in the A-EC cluster between the two groups. Notably, fluxes such as “Serine→ Pyruvate” and “Glutathione→ Glutamate” showed significant differences (*P* < 0.05), indicating a profound rewiring of energy metabolism and antioxidant metabolic fluxes in the A-ECs of sheep with different reproductive capabilities.

### Alterations in intercellular communication and ligand-receptor interactions between the HLS and LLS groups

CellChat analysis predicted group-dependent differences in the communication networks among ovarian cell populations. Globally, the total number of inferred interactions was slightly higher in the LLS group (556 pairs) compared to the HLS group (513 pairs), while the total interaction strength remained comparable (Fig. S3B). Thus, the higher interaction count in LLS ovaries indicates a broader set of predicted ligand–receptor pairs rather than globally stronger communication.

**Fig. 7:**
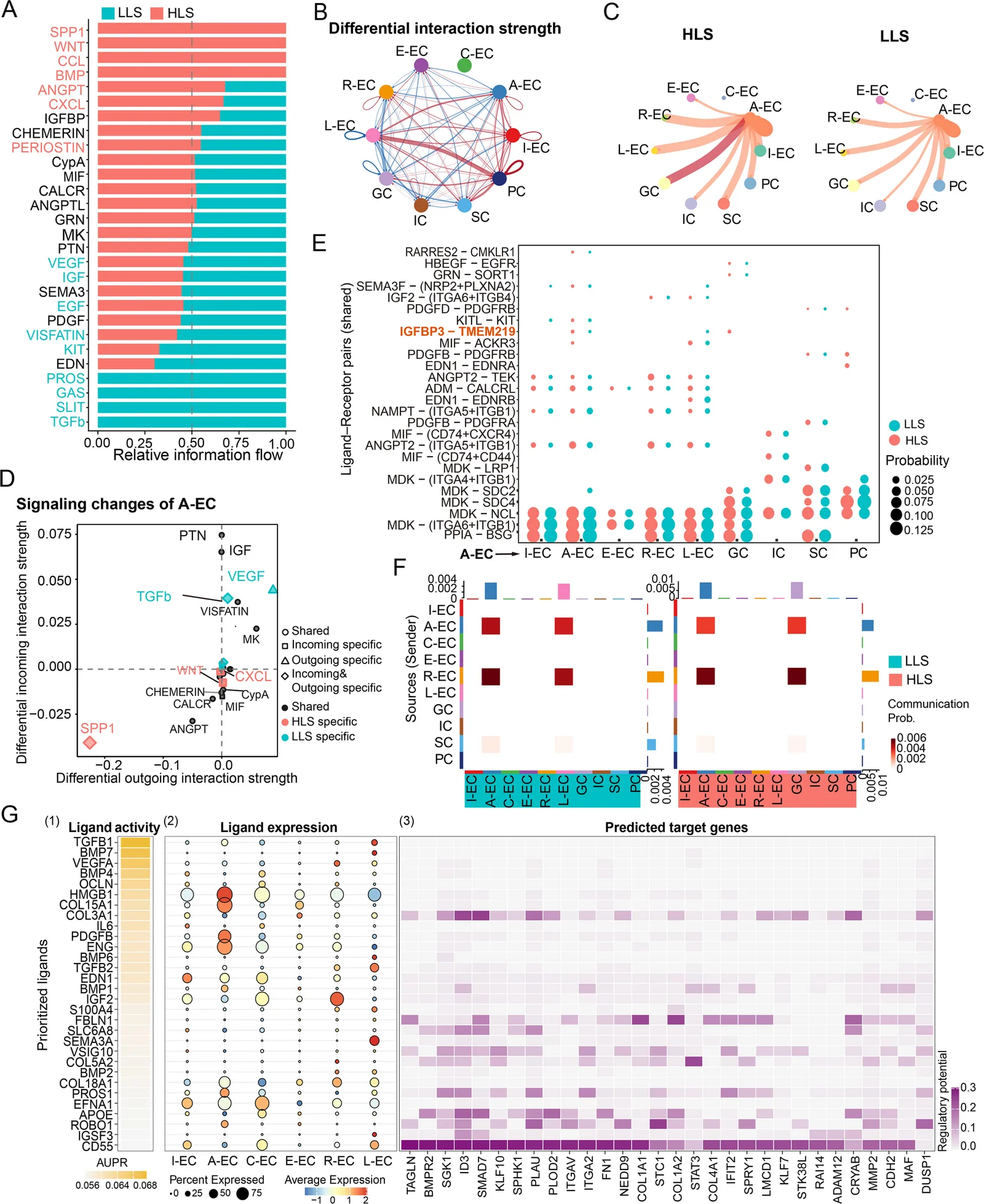
Cell–cell communication patterns associated with A-ECs in LLS and HLS ovaries. **A** The relative information flow of significant signaling pathways. **B** The interaction strength among different cell populations. **C** A-EC communication networks in HLS and LLS groups. Edge width represents the inferred interaction strength. **D** Differential outgoing and incoming signaling changes of A-ECs between HLS and LLS groups. Shared, HLS-specific and LLS-specific pathways are indicated by point color and shape. **E** Probability map of receptor-ligand pairs across different cell populations. Dot size indicates communication probability, and color denotes group identity. **F** Heatmaps showing communication probabilities of selected A-EC-related signaling interactions across sender and receiver cell populations in the IGFBP pathway. **G** Ligand prioritization analysis of A-EC-related signaling. (1) Ranked ligand activity based on AUPR scores. (2) Ligand expression across EC subtypes, with dot size indicating the percentage of expressing cells and color indicating average expression. (3) Predicted target-gene regulatory potential for prioritized ligands

Comparison of relative information flow revealed group-dependent differences in signaling pathway activity between HLS and LLS ovaries (Fig. 7A). WNT, CCL and BMP signaling showed greater relative information flow in the HLS group, whereas GAS, SLIT and TGFβ signaling were more prominent in the LLS group. Differential network analysis further identified the cell-population pairs showing increased or decreased predicted interaction strength between groups (Fig. 7B). When the analysis was restricted to A-ECs as sender cells, their predicted communication with multiple ovarian cell populations was generally stronger in HLS than in LLS ovaries (Fig. 7C).

We next examined which signaling pathways contributed to the altered A-EC communication pattern. Pathway-level comparison revealed group-dependent changes in both outgoing and incoming A-EC signaling, involving pathways such as PTN, IGF, VEGF, TGFβ, SPP1 and WNT (Fig. 7D). Analysis at the ligand–receptor level further identified specific pairs contributing to these pathway-level differences and highlighted IGFBP3–TMEM219 as a candidate interaction for subsequent investigation (Fig. 7E). Network analysis of the IGFBP pathway showed that the predicted communication changes were concentrated in specific sender–receiver combinations rather than uniformly distributed across all ovarian cell populations (Fig. 7F). Finally, NicheNet analysis was performed using the six EC subtypes as sender populations and GCs as the receiver population. This analysis prioritized EC-derived ligands according to their ability to explain transcriptional differences in GCs and linked candidate ligands, including TGFB1, BMP7 and VEGFA, to their predicted target genes (Fig. 7G). Together, these analyses identified pathway and cell-pair-specific changes in EC communication and provided the basis for selecting IGFBP3–TMEM219 for functional evaluation.

### Functional assessment of endothelial derived IGFBP3 in granulosa cells

**Fig. 8:**
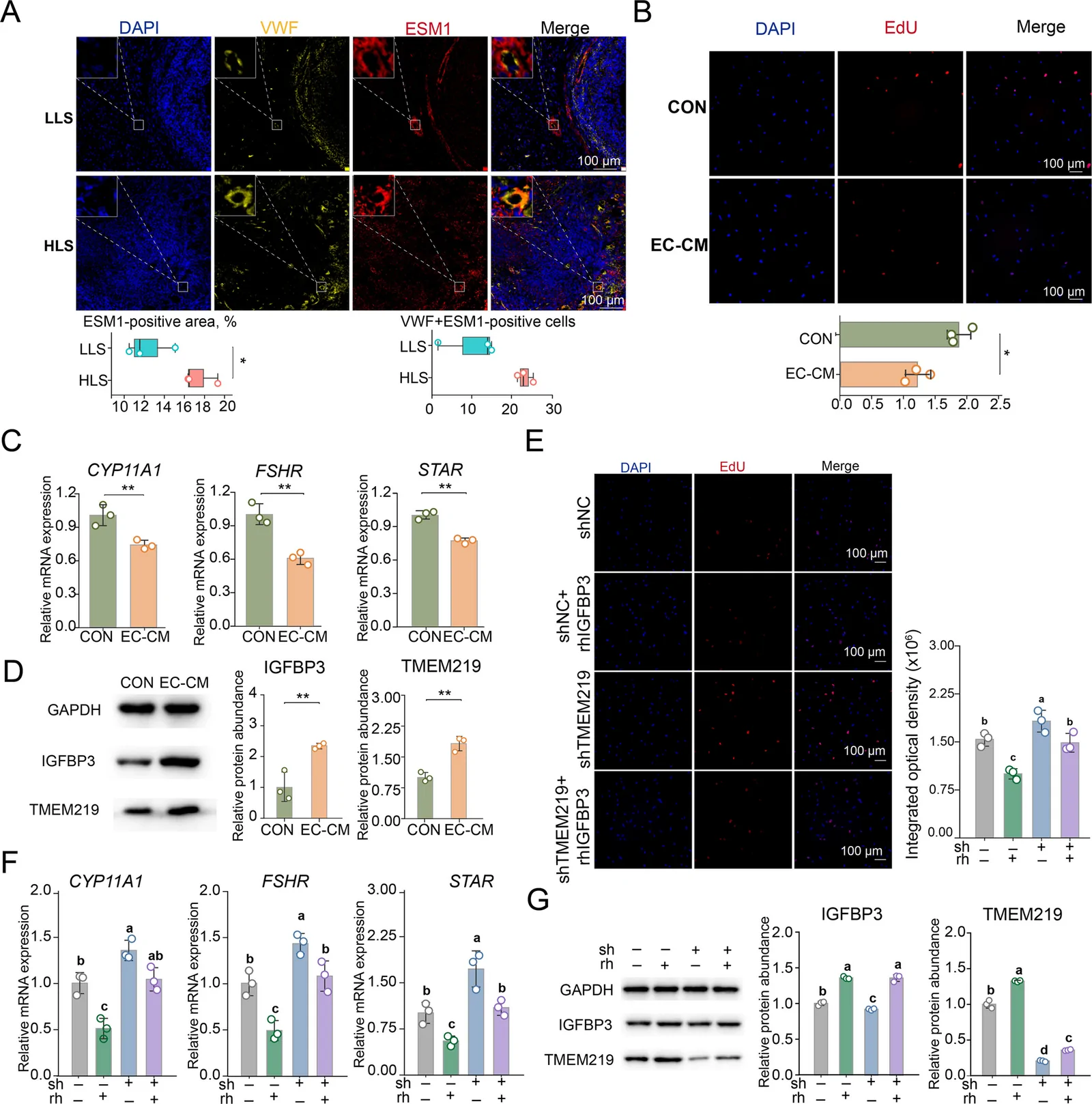
Histological and functional assessment of endothelial derived IGFBP3 in granulosa cells. **A** Representative immunofluorescence images of ESM1 and the endothelial marker VWF in ovarian sections from LLS and HLS Hu ewes, with quantification of the ESM1-positive area and VWF⁺ESM1⁺ cells. Boxes indicate the regions shown at higher magnification. **B** Representative EdU staining and quantification of granulosa cells. The box plot shows the relative ratio of EdU‑positive cells between the CON and EC‑CM groups. **C** Relative mRNA expression of *CYP11A1*, *FSHR* and *STAR* in granulosa cells following EC-CM treatment. **D** Representative Western blots and densitometric quantification of IGFBP3 and TMEM219 in granulosa cells. **E** Representative EdU staining and quantification in granulosa cells following TMEM219 knockdown and rhIGFBP3 treatment. **F** Relative mRNA expression of *CYP11A1*, *FSHR* and *STAR* in the four treatment groups following TMEM219 knockdown and rhIGFBP3 treatment. **G** Representative Western blots and densitometric quantification of IGFBP3 and TMEM219 following TMEM219 knockdown and rhIGFBP3 treatment. GAPDH was used as the loading control. In panels **E**–**G**, “sh” denotes TMEM219 knockdown and “rh” denotes rhIGFBP3 treatment. “ + ” and “ − ” indicate the presence or absence of the corresponding treatment. Scale bars, 100 μm. Different letters above bar indicate *P* < 0.05; ^*^P < 0.05, ^**^P < 0.01

To obtain experimental support for the endothelial heterogeneity and endothelial–granulosa cell communication predicted by the integrated analyses, we performed complementary tissue localization and cell based functional experiments. We first examined whether the ESM1⁺ EC population identified by single-cell analysis could be detected in ovarian tissue and whether its abundance differed between LLS and HLS ewes. Immunofluorescence staining showed that ESM1 was associated with VWF-positive vascular structures in Hu sheep ovaries (Fig. 8A). The ESM1-positive area was higher in HLS than in LLS ovaries (17.41% ± 1.68% vs. 12.41% ± 2.40%, *P* = 0.042). The number of VWF⁺ESM1⁺ cells was also higher in the HLS group (23.33 ± 2.02 vs. 10.17 ± 7.52 cells, *P* = 0.043), supporting the endothelial localization of ESM1 and its association with the HLS phenotype.

We next examined the expression pattern underlying the predicted IGFBP3–TMEM219 communication. Sample-level single-cell analysis showed numerically lower IGFBP3 expression in HLS than in LLS ewes, both across all endothelial cells and specifically within A-ECs. TMEM219 expression in granulosa cells was comparable between the two groups (*P* = 0.321) (Fig. S3D). Thus, the predicted communication difference was not attributable to a marked increase in ligand or receptor expression. Nevertheless, the lower mean IGFBP3 expression in HLS endothelial cells provided a directional basis for testing whether endothelial-derived IGFBP3 negatively regulates granulosa-cell activity.

We next examined the effects of endothelial-derived signals on primary ovine GCs using HOMEC-derived EC-CM and rhIGFBP3, thereby establishing a cross-species validation system. Exposure of ovine granulosa cells to EC-CM reduced EdU staining intensity compared with the control treatment (*P* = 0.013; Fig. 8B). EC-CM also decreased the relative mRNA expression of *CYP11A1*, *FSHR* and *STAR* (*P* = 0.009, 0.003 and < 0.001, respectively; Fig. 8C), while increasing cellular IGFBP3 and TMEM219 protein abundance (*P* = 0.008 and 0.002, respectively; Fig. 8D). These results indicate that factors released by endothelial cells alter proliferative activity and functional gene expression in granulosa cells.

We subsequently evaluated whether TMEM219 contributed to the effects associated with IGFBP3. In control-transfected granulosa cells, rhIGFBP3 reduced EdU intensity and the expression of *CYP11A1*, *FSHR* and *STAR* (Fig. 8E and F), consistent with the direction of the EC-CM response. TMEM219 knockdown alone increased these measurements, whereas the responses to rhIGFBP3 were attenuated after TMEM219 knockdown. Western blotting confirmed a marked reduction in TMEM219 protein abundance in the knockdown groups (Fig. 8G). Together, these results support IGFBP3–TMEM219 as a potential signaling route through which endothelial cells regulate granulosa-cell function.

## Discussion

Hu sheep are known for their high prolificacy, yet litter size remains variable even among ewes carrying the same *Fec*^*B*^ genotype, indicating that major genetic determinants do not fully explain individual reproductive performance [3, 5]. In this study, we examined whether this variation was also associated with the ovarian metabolic environment and its cellular organization. Ovarian metabolomics revealed differences in lipid metabolism between LLS and HLS ewes. Integration with single-cell transcriptomic data identified ECs as another important somatic cell population associated with these differences. In particular, ESM1⁺ A-ECs exhibited a distinct metabolic state in HLS ovaries. Validation at the tissue and cellular levels further supported the association of ESM1⁺ ECs with the HLS phenotype and showed that ECs derived signals influence GCs functional characteristics. Together, these findings suggest that litter-size variation in Hu sheep is associated with differences in the ovarian metabolic microenvironment and the functional heterogeneity of ovarian somatic cells. Importantly, our results further link these metabolic alterations to endothelial–granulosa cell communication, highlighting a potential role of endothelial regulation in shaping the ovarian environment. This study provides additional insights into how cellular and metabolic states may contribute to reproductive differences.

### Ovarian metabolic differences associated with litter-size variation in Hu sheep

Our metabolomic analysis provided an exploratory view of ovarian metabolic patterns associated with litter-size variation in Hu sheep. Although the candidate metabolites met the screening criteria of VIP > 1 and nominal *P* < 0.05, none remained statistically significant after BH correction (adjusted *P* = 0.171–0.590 for GC–MS and 0.200–0.690 for LC–MS; Additional file 2). These findings should therefore be interpreted as hypothesis-generating patterns rather than confirmed differences between the HLS and LLS groups. Among these exploratory candidates, L-carnitine showed higher abundance in HLS ovaries, whereas several SM species showed higher abundance in LLS ovaries. Lipid metabolism within ovarian follicles supports energy production, membrane remodeling and steroid synthesis, and its activity varies among follicular cell types and developmental stages [29]. L-carnitine transports long-chain fatty acyl groups into mitochondria and participates in cellular redox regulation. In an oxidative-stress model of human GCs, L-carnitine reduced ROS accumulation and apoptosis while preserving mitochondrial membrane potential and FSHR expression [30]. More directly relevant to sheep reproduction, Goldansaz et al. identified L-carnitine as a serum metabolite associated with ewe litter size [31]. Consistent with this association, our results identify higher ovarian L-carnitine as a metabolic characteristic of HLS ewes.

The higher abundance of several SM species in LLS ovaries represents a different aspect of lipid regulation. SMs are structural components of cell membranes and also provide substrates for the production of ceramide and sphingosine-1-phosphate (S1P). In GCs, activation of SM hydrolysis increased ceramide production and inhibited FSH-stimulated progesterone synthesis by reducing the expression of steroidogenic enzymes [32]. By contrast, a recent study in bovine GCs showed that S1P was required for the increase in cell viability induced by FSH, although it did not mediate the effect of FSH on estradiol synthesis [33]. These findings indicate that SM-derived metabolites can exert different effects on GC survival and steroidogenic activity. Consequently, the higher abundance of selected SM species in LLS ovaries may reflect changes in membrane lipid composition and in the balance of sphingolipid-related signaling, rather than a uniformly beneficial or adverse lipid state.

KEGG analysis provided additional pathway-level context for these metabolite differences. Consistent with these lipid-related differences, KEGG pathway analysis highlighted the enrichment of “fatty acid biosynthesis” and “β-alanine metabolism”, further supporting alterations in lipid related processes. Combined with these observations, the higher L-carnitine abundance in HLS ovaries and the accumulation of selected SM species in LLS ovaries define different but related features of ovarian lipid regulation. These metabolic differences provided the basis for our subsequent integration with single-cell transcriptomic data.

### Metabolic and functional heterogeneity of ovarian ECs in Hu sheep

Integrated transcriptomic and metabolomic analyses showed that the metabolic differences between LLS and HLS ovaries were not distributed uniformly across ovarian somatic cells. In addition to GCs, ECs emerged as another important cell population associated with these differences. This finding is consistent with the extensive vascular remodeling that accompanies the ovarian cycle [34]. In ruminants, angiogenesis is particularly active during follicular development and luteinization and depends on coordinated interactions among ovarian cell populations [35]. Dath et al. further showed that co-transplantation of ECs with stromal cells is essential for reconstructing a vascularized and structurally intact ovarian stroma in premature ovarian failure [36]. Adequate vascularization supports the delivery of oxygen, nutrients, hormones and metabolic substrates to the developing follicle. By contrast, disruption of angiogenesis can impair follicular growth and luteal function [37]. These observations indicate that ECs are active components of the ovarian microenvironment rather than passive vascular structures.

Our analysis highlighted two metabolite-associated genes, with cell-type-specific expression patterns. *DNAJB4* was mainly expressed in SCs and ECs and was expressed at lower levels in HLS ovaries. Although *DNAJB4* has previously been associated with reproductive and production traits [38, 39], its lower expression in HLS ovaries may indicate a negative association with prolificacy. However, its functional relevance remains to be established. On the other hand*, NDRG1* participates in endothelial stress responses, inflammation and vascular remodeling [40]. It has also been positively correlated with follicular angiogenesis markers [41, 42]. We found *NDRG1* to be highly expressed in ECs and significantly elevated in the HLS group, suggesting it supports high prolificacy by enhancing follicular angiogenesis. At the pathway level, HLS ECs showed greater enrichment of oxidative phosphorylation and glutathione metabolism. These pathways may support cellular energy requirements while maintaining redox balance. The higher expression of *NDRG1*, *GCLC* and *ATP5F1A* in HLS ovaries was consistent with this interpretation. These expression patterns further support an altered metabolic state in HLS ECs.

Given the limited understanding of EC subsets in sheep, we initially categorized these cells based on key differential markers. The resulting classification was supported by the distinct developmental trajectories observed in the pseudotime analysis. Crucially, we found that ESM1^+^ A-ECs are highly concordant with the key metabolic pathways highlighted by our metabolomic data. *ESM1* is enriched in endothelial tip cells and can enhance VEGF bioavailability, thereby influencing endothelial sprouting and vascular permeability [43]. It has also been implicated in angiogenic regulation through eNOS- and HIF-1α-related signaling [44]. Consistent with the single-cell analysis, ESM1 was localized to VWF-positive vascular structures in ovarian tissue, and ESM1-positive staining was higher in HLS ovaries. At the whole-EC level, oxidative phosphorylation and glutathione metabolism were enriched in the HLS group. Further subclustering showed that A-ECs exhibited group-dependent differences in these metabolic programs. These findings identify ESM1^+^ A-ECs as an endothelial population associated with ovarian metabolic variation. Nevertheless, further evidence is required to determine whether this population directly regulates follicular development or litter size.

### EC-GC communication and the candidate IGFBP3-TMEM219 pathway

The ovary is a highly heterogeneous organ that relies on the coordinated action of diverse cell populations to maintain follicular development and ovarian function. Among the identified cell types, GCs have been the most extensively investigated. GCs support follicular development, steroidogenesis and the acquisition of ovulatory competence [16, 45]. EC-derived signals may also contribute to follicular development. In sheep, FGF18 produced by thecal ECs acts on the granulosa layer, promotes GC apoptosis and restricts follicle growth [10]. Our cell-communication analysis predicted differences in EC-GC signaling between LLS and HLS ovaries. Although the LLS group contained more inferred ligand–receptor pairs, the overall interaction strength was comparable between groups. This pattern suggests that litter-size variation was associated with changes in the organization and pathway composition of the predicted communication network, rather than a global increase or decrease in ovarian cell–cell communication. Within the inferred ligand-receptor interactions, IGFBP3-TMEM219 was selected for functional validation. IGFBP3 expression showed a decreasing trend in HLS ECs and ESM1^+^ A-ECs, although the differences did not reach statistical significance. Because IGFBP3 is a secreted protein, this trend suggests that EC-derived IGFBP3 may represent a candidate paracrine signal involved in EC–GC cell communication.

Previous studies support an inhibitory role of IGFBP3 in ovarian function. In cultured human GCs, IGFBP3 dose-dependently inhibited IGF-I-stimulated estradiol production and also reduced the response to FSH [46, 47]. In human GCs, increased IGFBP3 expression during the ovulatory period coincides with reduced proliferative activity and altered IGF signaling [48]. Moreover, IGFBP3 inhibited gonadotropin-induced ovulation, oocyte maturation and estradiol production in ovaries [49]. In sheep, IGFBP3 expression also changes across different stages of follicular development [50]. Consistent with these studies, our functional experiments showed that IGFBP3 suppressed GC proliferation and steroidogenesis-related gene expression, supporting an inhibitory paracrine effect of IGFBP3 on sheep GC function. Together, these observations link IGFBP3 more directly to preovulatory follicular function than to post-fertilization embryo survival, although they do not establish an effect on ovulation rate in Hu sheep.

TMEM219 functions as a cell-surface receptor for IGFBP3 and has been implicated in the regulation of cell survival and tissue homeostasis [51, 52]. However, its involvement in ovarian GC responses to IGFBP3 has not previously been established. We found that TMEM219 was expressed in ovine GCs and that its knockdown partially attenuated the effects of rhIGFBP3 on GC proliferation and steroidogenesis-related gene expression. These results suggest that TMEM219 contributes to the response of GCs to IGFBP3, although it may not fully account for all of its effects. Combined with the expression trend observed in HLS ECs, these findings support IGFBP3-TMEM219 as a candidate EC-GC communication pathway regulating GC functional responses. However, whether this pathway contributes to litter-size variation in Hu sheep remains to be established.

Although these findings provide initial support for the proposed regulatory relationship, several aspects require further investigation given the limitations of the present study. To address the limited number of animals and potential body-weight-related variation, future studies will use larger experimental groups balanced for body weight and body condition, together with targeted metabolomics, to validate the identified metabolic patterns. Given the small sample size, the stability of supervised OPLS-DA models should also be interpreted cautiously, as the high VIP values observed for some LC–MS features may be influenced by model instability or overfitting. Ovulation rate and embryo survival will also be evaluated alongside litter-size records across multiple parities, allowing the observed ovarian differences to be related more precisely to specific reproductive processes. As ovine-specific pathway and ligand–receptor resources become available, they will be incorporated to refine the current cross-species inferences. Functional studies will further extend the present human EC-CM and rhIGFBP3 experiments to fully ovine systems using primary ovine ECs, ovine IGFBP3 and isolated ESM1⁺ A-ECs. These studies will further define the effects of IGFBP3–TMEM219 signaling on GC function and determine whether this pathway is associated with litter-size variation in Hu sheep.

## Conclusion

Our study indicates that litter-size variation in Hu sheep is accompanied by differences in the ovarian metabolic environment and somatic-cell organization. Metabolomic analysis identified exploratory patterns in ovarian carnitine and sphingolipid composition between HLS and LLS ewes. Further integration with single-cell transcriptomic data associated these metabolic differences with EC heterogeneity. Functional experiments showed that EC-derived and recombinant IGFBP3 suppressed GC proliferation and steroidogenesis-related gene expression, with TMEM219 contributing to these responses. Together, these findings associate EC heterogeneity with ovarian metabolic conditions relevant to follicular development and identify IGFBP3–TMEM219 as a functional EC–GC signaling pathway. Whether this pathway contributes to litter-size variation remains to be established. This study provides a cellular and metabolic perspective for understanding Hu sheep prolificacy and offers new directions for reproductive evaluation and breeding research.

## Supplementary Information

Additional file 1: Method S1. ScRNA dimensionality reduction, batch correction, and cell type annotation; Method S2. Pseudotime trajectory analysis using Monocle 2; Method S3. Intercellular communication analysis; Method S4. Multiplex immunofluorescence staining; Method S5. RNA extraction, reverse transcription and RT-qPCR; Method S6. Endothelial-cell conditioned medium and IGFBP3 quantification; Method S7. Primary isolation, culture and identification of granulosa cells; Method S8. Edu incorporation assay; Method S9. Western blotting; Method S10. Single-cell transcriptomic atlas of Hu sheep ovaries with litter size; Table S1. IGFBP3 concentrations in control and endothelial cell-conditioned medium samples; Table S2. IGFBP3 concentrations in endothelial cell-conditioned medium normalized to total cellular protein; Fig. S1. Isolation and identification of primary ovine granulosa cells; Fig. S2. Single-cell transcriptomic atlas and identification of major cell types in Hu sheep ovaries; Fig. S3. Validation and supporting analyses of endothelial metabolic features and endothelial–granulosa cell communication.

Additional file 2. Candidate differential metabolites identified by GC–MS and LC–MS, with variable importance in projection values, nominal *P* values, Benjamini–Hochberg-adjusted *P* values, fold changes and relative metabolite abundances.

Additional file 3. Uncropped gel and blot images.

## Data Availability

The datasets generated and/or analysed during the current study are available in the [NCBI gene expression omnibus database] repository (https://www.ncbi.nlm.nih.gov/geo/query/acc.cgi?acc=GSE233801, Accession Number: GSE233801).

## Abbreviations

A-EC: Angiogenic endothelial cell
ATP: Adenosine triphosphate
C-EC: Capillary endothelial cell
DEGs: Differentially expressed genes
EC: Endothelial cell
EC-CM: Endothelial cell-conditioned medium
ECM: Extracellular matrix
EdU: 5-Ethynyl-2′-deoxyuridine
EndMT: Endothelial-to-mesenchymal transition
E-EC: EndMT-like endothelial cell
Fec^B^: Booroola fecundity mutation
FSH: Follicle-stimulating hormone
GC: Granulosa cell
GC–MS: Gas chromatography–mass spectrometry
GO: Gene ontology
GSEA: Gene set enrichment analysis
HLS: High-litter-size group
HOMEC: Human ovarian microvascular endothelial cell
I-EC: Inflammatory endothelial cell
IC: Immune cell
IGF: Insulin-like growth factor
IGFBP: Insulin-like growth factor-binding protein
KEGG: Kyoto encyclopedia of genes and genomes
LC–MS: Liquid chromatography–mass spectrometry
LLS: Low-litter-size group
L-EC: Lymphatic endothelial cell
NES: Normalized enrichment score
OPLS-DA: Orthogonal partial least-squares discriminant analysis
PC: Perivascular cell
PCA: Principal component analysis
R-EC: Arterial Endothelial cell
rhIGFBP3: Recombinant human IGFBP3
RT-qPCR: Reverse-transcription quantitative PCR
S1P: Sphingosine-1-phosphate
SC: Stromal cell
scFEA: Single-cell flux estimation analysis
scRNA-seq: Single-cell RNA sequencing
shNC: Non-targeting short hairpin RNA control
shRNA: Short hairpin RNA
SMs: Sphingomyelins
TCA cycle: Tricarboxylic acid cycle
TGF-β: Transforming growth factor beta
UMAP: Uniform manifold approximation and projection
VEGF: Vascular endothelial growth factor
VIP: Variable importance in projection
