# Supplementary material for: Integrated single-cell and metabolomic profiling identifies ovarian metabolic differences and endothelial heterogeneity associated with litter-size variation in Hu sheep
Source: J Anim Sci Biotechnol. 2026 Oct 9;17:191. doi: 10.1186/s40104-026-01508-6 (PMC13651338; doi:10.1186/s40104-026-01508-6)
Supplement: Supplementary file 1 [file 40104_2026_1508_MOESM1_ESM.docx]

**Method S1 ScRNA dimensionality reduction, batch correction, and cell type annotation**

The quality-controlled cells were normalized and scaled using Seurat, followed by Principal Component Analysis (PCA). To mitigate batch effects across different samples, Harmony correction was applied to the PCA embeddings. The Harmony-corrected dimensions were then utilized for graph-based neighbor construction, clustering, and Uniform Manifold Approximation and Projection (UMAP) visualization.

Initial cell type annotation was performed based on cluster-specific differentially expressed genes (identified via FindAllMarkers) and canonical marker genes from the literature and databases.

**Method S2 Pseudotime trajectory analysis using Monocle 2**

Trajectory analysis was performed using Monocle 2. First, a CellDataSet object was constructed by converting the expression matrix into a sparse matrix (newCellDataSet). Normalization and dispersion estimation were carried out using estimateSizeFactors and estimateDispersions, respectively. Genes with low expression were filtered out using detectGenes(min_expr=0.1), retaining only those detected in at least 0.5% of the cells. Dimensionality reduction was performed using the DDRTree algorithm (reduceDimension, max_components=2, method="DDRTree"), followed by cell ordering along the pseudotime trajectory using orderCells(). Genes showing dynamic expression changes along pseudotime were clustered and visualized using plot_pseudotime_heatmap(). To identify branch-dependent gene expression changes, Branched Expression Analysis Modeling (BEAM) was applied at key branch points. The resulting branch-dependent genes were visualized using the ClusterGVis R package.

**Method S3 Intercellular communication analysis**

For CellChat analysis, a CellChat object was created using the CellChatDB.human ligand-receptor database as the reference resource. Standard preprocessing steps were executed, including gene and interaction filtering (subsetData, identifyOverExpressedGenes, identifyOverExpressedInteractions). Communication probabilities were computed (computeCommunProb), and interactions involving few cells were filtered (filterCommunication). Pathway-level probabilities and aggregated networks were then generated (computeCommunProbPathway, aggregateNet). Network centrality metrics were calculated (netAnalysis_computeCentrality) to identify key sender/receiver cell populations and compare communication patterns between the HLS and LLS groups.

For NicheNet analysis, the six endothelial cell subpopulations were defined as sender cells, and granulosa cells were defined as receiver cells. Differentially expressed response genes in receiver cells (HLS vs. LLS) were identified. Ligand activity was predicted based on the NicheNet weighted network and ligand-target matrix. Potential key ligands were prioritized, and the “ligand-receptor-target gene” regulatory axis was extracted to elucidate the underlying molecular mechanisms.

**Method S4 Multiplex immunofluorescence staining**

Paraffin-embedded ovarian sections from HLS and LLS Hu ewes were used to examine the vascular localization of ESM1 (*n* = 3 animals per group). Sections were deparaffinized in three changes of xylene for 15 min each and rehydrated through absolute ethanol, 85% ethanol and 75% ethanol before washing in distilled water. Antigen retrieval was performed in citrate buffer by microwave heating for 20 min. After cooling, the sections were washed three times in phosphate-buffered saline (PBS) for 5 min each. Endogenous peroxidase activity was blocked with 3% hydrogen peroxide for 25 min at room temperature, followed by blocking with 3% bovine serum albumin for at least 30 min. Sections were sequentially incubated at 4℃ overnight with antibodies against VWF (HUABIO, HA722833; 1:100) and ESM1 (Affinity Biosciences, DF14935; 1:100). After each primary-antibody incubation, sections were incubated with horseradish peroxidase-conjugated goat anti-rabbit secondary antibody (Servicebio, GB23303; 1:100). Fluorescence signals were developed using tyramide signal amplification reagents (1:500), with antibody stripping and repeated antigen retrieval between staining cycles. Nuclei were counterstained with DAPI for 10 min, and the sections were mounted using antifade mounting medium.

Whole-slide images were acquired using an Olympus VS200 scanner. Two non-overlapping fields at ×200 magnification were included for each animal in the final quantitative dataset. The ESM1-positive area was quantified using HALO software, and VWF/ESM1 double-positive cells were counted using Image-Pro Plus. Field-level measurements were averaged to obtain one value per animal before statistical analysis; microscopic fields were not treated as independent biological replicates.

**Method S5 RNA extraction, reverse transcription and RT-qPCR**

The same RNA extraction, reverse-transcription and RT-qPCR procedures were used for all tissue- and cell-based validation experiments. Total RNA was isolated from ovarian tissues or cultured cells using a Total RNA Extraction Kit (Biosharp, BL1365A) with RNA Extraction Auxiliary Reagent (Biosharp, BL1665A). Genomic DNA was removed using gDNA Eraser, and cDNA was synthesized using the PrimeScript RT Reagent Kit with gDNA Eraser (Takara Bio, RR047A). Quantitative PCR was performed on a QuantStudio 3 Real-Time PCR System using TB Green Premix Ex Taq II FAST qPCR reagent (Takara Bio, CN830A). GAPDH was used as the reference gene.

**RT-qPCR reaction mixture and amplification conditions:**

RT-qPCR was performed in a total reaction volume of 25 μL. The reaction mixture was prepared as follows:

| **Component** | **Volume, μL** |
| --- | --- |
| TB Green Premix Ex Taq II Fast qPCR (2×) | 12.5 |
| Forward primer (10 μmol/L) | 1.0 |
| Reverse primer (10 μmol/L) | 1.0 |
| cDNA template | 2.0 |
| ddH₂O | 8.5 |
| Total volume | 25.0 |

The amplification programme consisted of initial denaturation at 95 °C for 1 min, followed by 45 cycles of denaturation at 95 °C for 10 s, annealing at 55 °C for 30 s, and extension at 72 °C for 10 s. Fluorescence signals were acquired at the end of each extension step.

| **Step** | **Temperature** | **Time** | **Cycles** |
| --- | --- | --- | --- |
| Initial denaturation | 95 ℃ | 1 min | 1 |
| Denaturation | 95 ℃ | 10 s | 45 |
| Annealing | 55 ℃ | 30 s | 45 |
| Extension and fluorescence acquisition | 72 ℃ | 10 s | 45 |

**Method S6 Endothelial-cell conditioned medium and IGFBP3 quantification**

ovarian microvascular endothelial cells (HOMECs; Cellverse, hum-icell-f050) were used to generate endothelial-cell conditioned medium (EC-CM). After the cells had reached the predefined confluence, the culture medium was removed, the cells were washed with phosphate-buffered saline, and serum-free medium was added. Conditioned medium was collected after 48 h, clarified by centrifugation. Serum-free medium processed in parallel served as the medium control. IGFBP3 concentrations in the serum-free medium control and HOMEC-derived EC-CM were measured using a Human IGFBP3 ELISA Kit (Shanghai Zhuocai Biotechnology, ZC-35444; 48 tests). Standards were prepared at 3.75, 7.5, 15, 30, 60 and 120 ng/mL. Standards and samples (50 μL per well) were added to the assay plate, followed by 100 μL of horseradish peroxidase-conjugated detection antibody, except in the blank wells. The plate was sealed and incubated at 37 ℃ for 60 min. After five wash cycles, 50 μL each of substrates A and B were added, and the plate was incubated at 37 ℃ in the dark for 15 min. The reaction was stopped with 50 μL of stop solution, and absorbance was measured at 450 nm within 10 min. Sample concentrations were calculated from the standard curve after subtraction of the blank absorbance and correction for dilution.

To standardize EC-CM batches, total protein was measured in the corresponding HOMEC lysates using a BCA assay. The IGFBP3 concentration in each EC-CM preparation was normalized to the total protein content of the same endothelial-cell culture and expressed as ng IGFBP3 per mg total protein. Only EC-CM preparations that met the predefined protein-normalized quality-control criterion were used in subsequent granulosa-cell experiments. The serum-free medium control was used to assess assay and medium background and was not treated as a cell-derived sample.

IGFBP3 concentrations were higher in EC-CM than that in control samples (11.174 ± 2.646 vs. 2.946 ± 0.312 ng/mL; *n* = 3 per group; one-way ANOVA, *P* = 0.0059). After normalization to total protein, the mean IGFBP3 content of the EC samples was 1.983 ± 0.521 ng/mg total protein.

**Table S1** IGFBP3 concentrations in control and EC-CM samples

| **Group** | ***n*** | **Replicate values, ng/mL** | **Mean ± SD, ng/mL** | **ANOVA *P* value^a^** |
| --- | --- | --- | --- | --- |
| Control | 3 | 2.812, 3.303, 2.723 | 2.946 ± 0.312 |  |
| EC-CM | 3 | 13.762, 8.473, 11.288 | 11.174 ± 2.646 | 0.0059 |

^a^*P* value represents the overall comparison between the Control and EC-CM groups

**Table S2** IGFBP3 concentrations normalized to total protein in EC samples

| **Group** | ***n*** | **IGFBP3, ng/mg** |
| --- | --- | --- |
| EC | 3 | 1.983**±**0.521 |

**Method S7 Primary isolation, culture and identification of granulosa cells**

GCs were isolated and cultured as previously described. Briefly, ovarian tissue was rinsed two to three times in pre-warmed sterile saline containing 1% penicillin-streptomycin and placed in a culture dish containing modified Dulbecco’s phosphate-buffered saline (mDPBS). Follicles were punctured with a sterile syringe, and the released follicular fluid was collected and centrifuged at 1000 r/min for 5 min. After washing with mDPBS, the cell pellet was resuspended in complete culture medium and incubated at 37 ℃ in a humidified atmosphere containing 5% CO_2_.

Through microscopic observation of cell morphology, it was found that the primary cells cultured in this study were slender spindle-shaped cells, which were consistent with the morphological characteristics of granulosa cells under the microscope (Fig. S1A). At the same time, the positive immunofluorescence results of the marker protein FSHR of granulosa cells (Fig. S1B) confirmed that the primary ovarian granulosa cells of Hu sheep were isolated and cultured in this study.

**Fig. S1** Isolation and identification of primary granulosa cells from Hu sheep ovaries. **A** Microscopic observation of cell morphology, scale bar: 1,000μm. **B** Immunofluorescent identification of granulosa cells, scale bar: 4×, 1,000μm; 10×, 400μm. Blue, DAPI; Green, FSHR; MERGE, composite

**Method S8 EdU incorporation assay**

Granulosa-cell proliferative activity was assessed using an EdU incorporation assay according to the manufacturer’s instructions. After the indicated EC-CM or shRNA/rhIGFBP3 treatment, cells were incubated with EdU reagent, fixed and subjected to fluorescent detection. Nuclei were counterstained with DAPI. Images were acquired using identical exposure and acquisition settings for all groups within the same experiment. EdU signal was quantified as integrated optical density, and the percentage of EdU-positive cells was additionally calculated during the EC-CM experiment. Three independent biological replicates were analysed per group, with the same number of microscopic fields quantified for each replicate.

**Method S9 Western blotting**

Granulosa-cell pellets were lysed in pre-cooled RIPA buffer (Bioss, C5029) containing protease inhibitor and 1 mM PMSF. Approximately 100 μL of lysis buffer was used per 10⁶ cells. Cell suspensions were sonicated for 3 s followed by a 10-s pause for three to five cycles and incubated on ice for 30 min, with gentle mixing every 10 min. Lysates were centrifuged at 12,000 r/min for 10 min at 4 °C, and the supernatants were collected. Protein concentrations were determined using a BCA Protein Assay Kit (Biosharp, BL521C). Samples were mixed with 5× loading buffer and denatured at 95 °C for 10 min.

Proteins were separated on 10% SDS–PAGE gels at 100 V and transferred to 0.45-μm PVDF membranes at 200 mA for 1–2 h. Membranes were blocked in 5% non-fat milk in TBST for 2 h and incubated with primary antibodies at 4 °C overnight. The primary antibodies were IGFBP3 (Proteintech, 10189-2-AP; 1:2,000), TMEM219 (Ab-mart, M030834; 1:2,000) and GAPDH. GAPDH was detected using Proteintech 60004-1-Ig (1:200,000) in the EC-CM experiment and Abclonal AC033 (1:200,000) in the four-group shRNA/rhIGFBP3 experiment. After three 10-min washes in TBST, membranes were incubated for 2 h at room temperature with the appropriate horseradish peroxidase-conjugated secondary antibody (Abclonal AS014 or AS003; 1:8,000). Signals were developed using enhanced chemiluminescence and captured using a Tanon 5200 Multi imaging system. Band integrated optical density was quantified using Gel-Pro Analyzer, normalized to GAPDH, and expressed relative to the designated control group.

**Method S10 Single-cell transcriptomic atlas of Hu sheep ovaries with litter size**

To investigate the cellular heterogeneity underlying reproductive performance, we performed scRNA-seq analysis on ovarian tissues from HLS and LLS groups. After quality control, 20,540 cells from the LLS group and 21,182 cells from the HLS group were retained for downstream analysis. The single-cell data were corrected for batch effects, followed by dimensionality reduction and cell type identification for downstream analysis. Unsupervised clustering using Uniform Manifold Approximation and Projection (UMAP) initially revealed 20 distinct cell clusters based on global gene expression patterns (Fig. S2A).

Based on the expression of canonical marker genes, these clusters were annotated into five major cell lineages: Endothelial cells, Granulosa cells, Immune cells, Ovarian stromal cells, and Perivascular cells (Fig. S2B). The validity of these annotations was confirmed by the specific expression of known lineage markers (Fig. S2C). Specifically, Endothelial cells were defined by high expression of CDH5, CD34, and FLI1; Granulosa cells expressed follicular markers such as AMH, FSHR, and FST; Stromal cells were enriched for PDGFRA, DCN, and TCF21; Perivascular cells were characterized by RGS5 and MCAM; and Immune cells were identified by PTPRC (CD45) and CD3G.

The five major ovarian cell types accounted for 100% of cells in each group. In the LLS group, endothelial cells, granulosa cells, immune cells, ovarian stromal cells, and perivascular cells accounted for 50.52%, 10.42%, 9.17%, 22.95%, and 6.94% of cells, respectively. In the HLS group, the corresponding proportions were 41.55%, 10.61%, 7.28%, 33.05%, and 7.51%, respectively. Thus, endothelial and immune cells were proportionally higher in LLS ovaries, whereas ovarian stromal and perivascular cells were proportionally higher in HLS ovaries; granulosa-cell proportions were comparable between groups.

We further characterized the transcriptional signatures of each cell type. The heatmap (Fig. S2F) and differential expression analysis (Fig. S2E) demonstrated that each cell population possessed a unique gene expression profile. For instance, Endothelial cells specifically expressed VWF and ADGRL4, while Granulosa cells were enriched for KCNK12 and CRABP2, confirming the accuracy of our cell type classification.

**Fig. S2** Single-cell transcriptomic atlas and cell type identification in Hu sheep ovaries. **A** Uniform Manifold Approximation and Projection (UMAP) visualization of single cells isolated from sheep ovarian tissues. **B** Annotated UMAP plot identifying five major cell lineages. **C** Dot plot illustrating the expression patterns of canonical marker genes used for cell type identification. The color intensity represents the average expression level, and the dot size indicates the percentage of cells expressing the marker within each cluster. **D** Stacked bar chart comparing the cellular composition and relative proportions of each cell type between the Low Litter Size (LLS) and High Litter Size (HLS) groups. **E** Scatter plot visualizing the distribution of differentially expressed genes (DEGs) across the five cell types. The *y*-axis represents the average log_2_ Fold Change. Purple dots indicate significantly differentially expressed genes (adj *P* < 0.01), while green dots indicate non-significant genes. **F** Heatmap displaying the expression profiles of the top cell-type-specific marker genes across the five identified clusters. The color scale represents the Z-score normalized expression values

**Fig. S3** Validation and supporting analyses of endothelial metabolic features and endothelial–granulosa cell communication. **A** RT–qPCR validation of *NDRG1*, *GCLC* and *ATP5F1A* expression in ovarian tissues from low-litter-size (LLS) and high-litter-size (HLS) Hu ewes. **B** Total number and overall strength of predicted ligand–receptor interactions in the LLS and HLS groups. **C** Differential interaction network among endothelial subtypes and other ovarian somatic cell populations. Red edges indicate interactions increased in HLS relative to LLS, whereas blue edges indicate decreased interactions; edge width represents the magnitude of the difference. **D** Sample-level expression of IGFBP3 in all endothelial cells and A-ECs and of TMEM219 in granulosa cells. Expression is presented as log_2_ (CPM + 1). **E** RT-qPCR screening of three shRNAs targeting *TMEM219* in primary ovine granulosa cells. shTMEM219#1 was selected for subsequent functional experiments. Data in **A**, **D** and **E** are presented as the mean ± SD of three biological replicates, with individual values shown. In **E**, each shRNA group was compared with the shNC group. ns, not significant; ^*^*P* < 0.05, ^**^*P* < 0.01 and ^***^*P* < 0.001. GC, granulosa cells; IC, immune cells; SC, stromal cells; PC, perivascular cells; EC, endothelial cells. A-EC, C-EC, E-EC, I-EC, L-EC and R-EC denote the corresponding endothelial subtypes
