# Supplementary material for: Integrated single-cell and metabolomic profiling identifies ovarian metabolic differences and endothelial heterogeneity associated with litter-size variation in Hu sheep
Source: J Anim Sci Biotechnol. 2026 Oct 9;17:191. doi: 10.1186/s40104-026-01508-6 (PMC13651338; doi:10.1186/s40104-026-01508-6)
Supplement: Supplementary file 2 [file 40104_2026_1508_MOESM3_ESM.docx]

EC-CM1

CON1

EC-CM1

CON1

EC-CM1

CON1

**GAPDH**

**IGFBP3**

41-52 kDa

**TMEM219**

31 kDa

**Order of Western blot bands**:

shNC shNC+ rhIGFBP3 shTMEM219 shTMEM219+ rhIGFBP

N2

N1

N3

**GAPDH**

**IGFBP3**

41-52 kDa

**TMEM219**

31 kDa
